# O-GlcNAc modification differentially regulates microtubule binding and pathological conformations of tau isoforms *in vitro*

**DOI:** 10.1016/j.jbc.2025.108263

**Published:** 2025-02-03

**Authors:** Mohammed M. Alhadidy, Paul M. Stemmer, Nicholas M. Kanaan

**Affiliations:** 1 Department of Translational Neuroscience, College of Human Medicine, Michigan State University, Grand Rapids, Michigan, United States; 2 Neuroscience Program, Michigan State University, East Lansing, Michigan, United States; 3 Institute of Environmental Health Sciences, Wayne State University, Detroit, Michigan, United States; 4 Department of Pharmaceutical Sciences, College of Pharmacy and Health Sciences, Wayne State University, Detroit, Michigan, United States

**Keywords:** aggregation, Alzheimer’s disease, filaments, microtubules, O-glycosylation, oligomers, phosphatase-activating domain, posttranslational modification, protein conformation, protein misfolding, recombinant protein, tau, tauopathy

## Abstract

Tau proteins undergo several posttranslational modifications in physiological and disease conditions. In Alzheimer’s disease, O-GlcNAcylation modification of serine/threonine (S/T) residues in tau is reduced. In mouse models of tauopathy, O-GlcNAcase inhibitors lead to increased O-GlcNAcylation and decreased filamentous aggregates of tau. However, various nonfilamentous tau conformations, linked to toxicity and neurodegeneration in tauopathies, involve processes like oligomerization, misfolding, and greater exposure of the phosphatase-activating domain in the amino terminus of tau. Additionally, it is becoming clearer that posttranslational modifications may differently regulate tau pathobiology in an isoform-dependent manner. Therefore, it is crucial to investigate the effects of O-GlcNAcylation on nonfilamentous conformations of both the four-repeat (4R, *e.g.*, hT40) and three-repeat (3R, *e.g.*, hT39) tau isoforms. In this study, we assessed how O-GlcNAcylation impacts pathological tau conformations of the longest 4R and 3R tau isoforms (hT40 and hT39, respectively) using recombinant proteins. Mass spectrometry showed that tau is modified with O-GlcNAc at multiple S/T residues, primarily in the proline-rich domain and the C-terminal region. O-GlcNAcylation of hT40 and hT39 does not affect microtubule polymerization but has opposite effects on hT40 (increases) and hT39 (decreases) binding to preformed microtubules. Although O-GlcNAcylation interferes with forming filamentous hT40 aggregates, it does not alter the formation of pathological nonfilamentous tau conformations. On the other hand, O-GlcNAcylation increases the formation of pathological nonfilamentous hT39 conformations. These findings suggest that O-GlcNAcylation differentially modulates microtubule binding and the adoption of pathological tau conformations in the longest 4R and 3R tau isoforms.

Tauopathies are a group of neurodegenerative diseases, which includes Alzheimer’s disease (AD), corticobasal degeneration (CBD), progressive supranuclear palsy (PSP), Pick’s disease (PiD), and chronic traumatic encephalopathy, among others (1, 2, 3). Each of these diseases is characterized by the accumulation of pathological forms of the tau protein, a microtubule-associated protein (1, 2, 3, 4). Tau is normally expressed in the brain primarily as six main isoforms derived from alternative splicing that generates three proteins containing four microtubule-binding repeats (MTBRs; 4R isoforms) or three proteins containing three MTBRs (3R isoforms) and either 0, 1, or 2 N-terminal exons (5, 6). Interestingly, the hallmark tau pathologies are comprised of different tau isoforms with some being mixed (AD and chronic traumatic encephalopathy), primarily 3R isoforms (PiD), or primarily 4R isoforms (PSP and CBD) (1, 7). Despite the long-recognized tau-based pathologies in these diseases, the factors influencing the pathological misfolding, oligomerization, and filament aggregation of tau require further investigation.

Many factors likely impact the adoption of pathological conformations by tau including mutations (*e.g.,* P301L), protein–protein interactions (*e*.*g*., heat shock protein 90 and histone deacetylase 6), and PTMs (6). In fact, tau is well known for its susceptibility to undergo several PTMs in normal physiology as well as pathology associated with disease (6, 8, 9, 10). Certain PTMs are common among tauopathies and sufficient to induce pathological conformations in tau (11). The bulk of studies on tau PTMs were focused on phosphorylation and acetylation. While these are prominent tau PTMs involved in regulating its physiology and pathophysiology (10, 12), there are several other tau PTMs (*e*.*g*., glycosylation, SUMOylation, polyamination, methylation, nitration, *etc.*) (6) whose impacts on tau are understudied.

Modification of tau with O-GlcNAc is a PTM that involves the incorporation of GlcNAc onto serine/threonine (S/T) residues of proteins (13, 14). Of note, AD patients show regional hypometabolism in the brain that predicts future decline in cognitive and memory functions (15, 16, 17, 18, 19). The O-GlcNAcylation of tau is lower in the AD brain than brains of nondemented individuals (20, 21). These findings, among others, instigated an interest in the role played by O-GlcNAcylation in regulating tau pathobiology (22). In fact, O-GlcNAcylation of tau at S400 reduces the rate and extent of heparin-induced aggregation *in vitro* (23, 24). Furthermore, tau modification with O-GlcNAc is inversely related to its phosphorylation as demonstrated in studies that utilized recombinant tau proteins, cell lines, rat brain slices, mouse brains, and AD tissue (20, 21, 25, 26). Administering O-GlcNAcase (OGA) inhibitors (*i*.*e*., drugs that inhibit cleavage of O-GlcNAc from proteins) in WT Sprague-Dawley rats (27), rTg4510 mice (28, 29, 30), and JNPL3 mice (23) enhanced tau O-GlcNAcylation while reducing its phosphorylation. In animal models of tauopathy, treatment with Thiamet G, an OGA inhibitor, reduced neuronal loss, prevented brain atrophy, and inhibited tau aggregation (23, 28, 29).

Our understanding of the nonfilamentous misfolding and multimerization (*i*.*e*., oligomerization) of tau in pathological conditions has progressed over the years (6). Although cryo-EM highlights distinct tau filament core conformations in different tauopathies (31, 32, 33, 34, 35), growing evidence points to common conformational changes of tau into nonfilamentous species associated with toxicity (6, 11, 36, 37, 38, 39, 40, 41, 42, 43, 44, 45). One critical conformational change that arises early in disease conditions is tau oligomerization (36, 37, 43, 46). Tau oligomerization is linked to neurodegeneration and several forms of cellular dysfunction, including disruptions in intracellular transport, protein degradation, synaptic activity, and cellular energy regulation (40). Immunotherapies targeting tau oligomers demonstrated efficacy in reducing neurodegeneration without affecting tangle-like pathology in tauopathy mouse models (47, 48, 49, 50). Additionally, tau may adopt conformations that excessively expose the phosphatase-activating domain (PAD) in its extreme N terminus (amino acids 2–18) (51). Aberrant PAD exposure is associated with various pathogenic tau modifications, including oligomerization, mutations, and PTMs (46, 51, 52). For example, phosphomimetics at T175, S205, or S199/S202/T205, and tau mutations (*e*.*g*., P301L) induce axonal transport impairment through a PAD-dependent mechanism involving a protein phosphatase 1 and glycogen synthase kinase 3 β signaling pathway (51, 53, 54, 55, 56, 57). Early disease stages also are marked by tau adopting Alz50 or MC1 conformations [named for the antibodies used to detect them (58, 59, 60)] where its N terminus folds over the MTBR region (11, 59). This conformational shift is thought to represent an early marker along the path of tau aggregation.

Some specific PTMs are known to impact these misfolded and oligomeric forms of tau. For example, phosphorylation at the AT8 epitope colocalizes with PAD exposure and oligomeric tau in AD, and phosphomimetics at sites within the AT8 epitope causes PAD exposure and impair axonal transport (46, 51, 55, 61, 62, 63). However, whether O-GlcNAcylation affects the adoption of these abnormal conformational states of tau that occur early in disease conditions remains unknown. To this end, we produced recombinant O-GlcNAc modified 2N4R (Glc hT40) and 2N3R (Glc hT39) tau isoforms in bacterial cells by cotransformation with O-GlcNAcylation machinery. A set of biochemical and biophysical assays were employed to determine the impact of O-GlcNAcylation on tau’s interaction with microtubules, as well as tau aggregation and adoption of pathological conformations. Our results suggest that tau O-GlcNAcylation does not interfere with MT polymerization but may differentially regulate MT binding in a tau isoform dependent fashion. The data also suggest that O-GlcNAcylation differentially affects the formation of pathological tau conformations in an isoform-dependent manner. Taken together, this work identified isoform-dependent effects of O-GlcNAcylation on tau physiological function and the formation of pathogenic tau species. Further investigation of the role played by O-GlcNAcylation in tauopathies and potential O-GlcNAc-based therapeutics is warranted.

## Results

### Cotransformation of bacterial cells with tau and OGT introduces O-GlcNAc modifications on serine and threonine residues of tau

**Figure 1:**
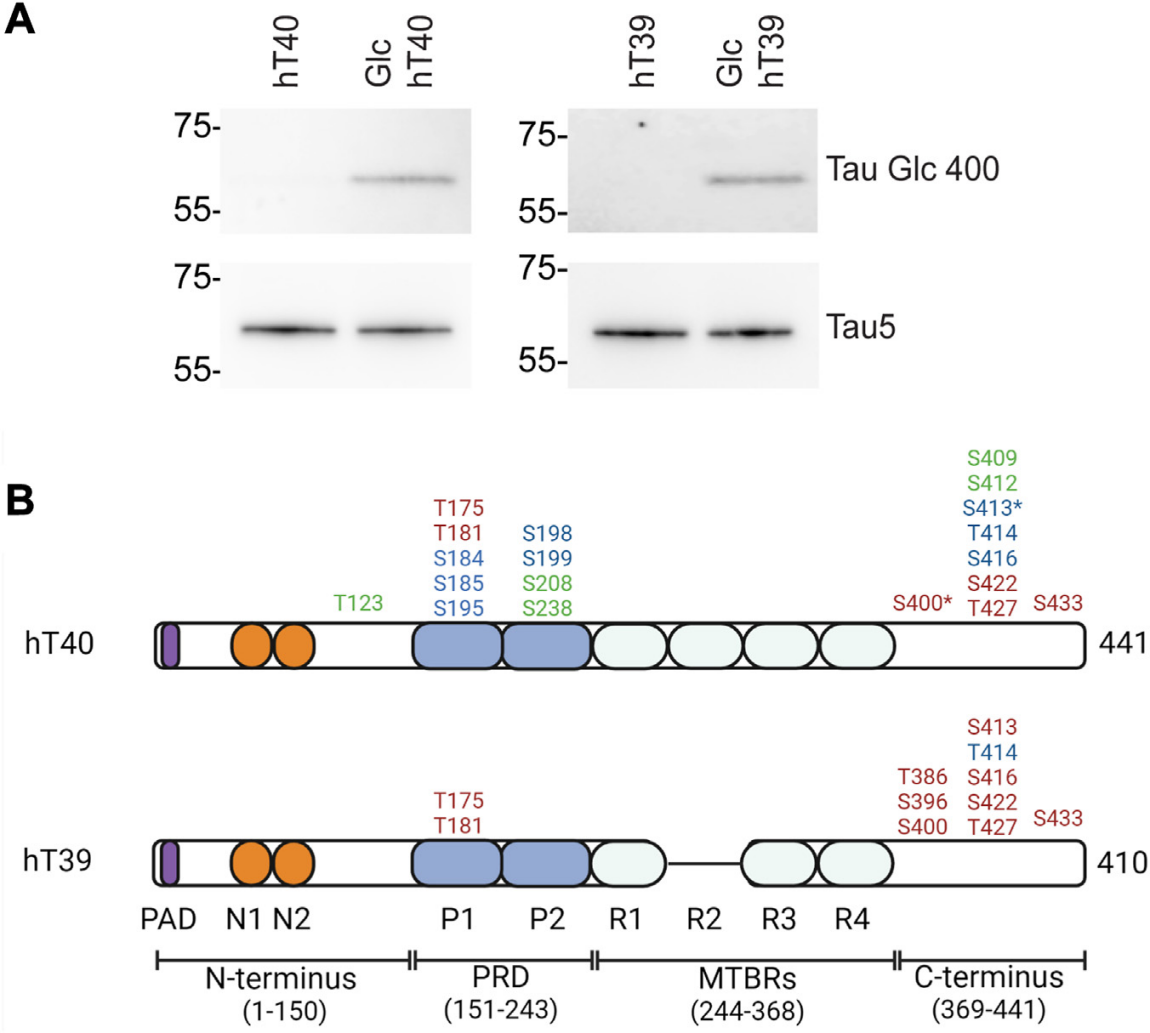
**Identification of S/T residues on tau modified with O-GlcNAc.***A*, representative Western blot of hT40 (*left blots*) and hT39 (*right blots*) proteins probed with the anti-O-linked-N-acetyl β-d-N-glucosamine (O-GlcNAc)-modified Ser400 Tau (*upper blots*) and Tau5 (total tau, *lower blots*) antibodies. *B*, serine (S) and threonine (T) residues modified with O-GlcNAc on tau as detected by mass spectrometry. Residues are numbered according to the sequence of full-length tau (hT40, 2N4R, 441 amino acids). Residues highlighted in *red* are confirmed modification sites in our study. For some O-GlcNAc sites, site-specific information could not be determined with high confidence (in *blue*). Residues highlighted in *green* represent GlcNAc modification sites reported in previous studies but not in our study. *Asterisk* (∗) represents modification sites common between our study and previous studies. GlcNAc sites are concentrated within the proline-rich domain (PRD) and C terminus. Notably, the microtubule-binding repeats (MTBRs) in hT40 and hT39 were not modified by O-GlcNAc. *Panel B* created with BioRender.com.

To confirm the modification status of the final protein preparations, western blotting for O-GlcNAc Ser400 tau and total tau was employed. Unmodified hT40 and hT39 did not label with the anti-Tau O-GlcNAc Ser400 antibody, while the modified hT40 and hT39 were labeled by the anti-Tau O-GlcNAc Ser400 antibody (Fig. 1*A*). Both unmodified and modified tau proteins showed similar reactivity to the Tau5 antibody, a pan-tau antibody (Fig. 1*A*).

Availability of Glc tau-specific antibodies is limited to the Ser400 modification site antibody. To more rigorously identify which S/T residues are modified throughout the hT40 and hT39 proteins, mass spectrometry (MS) analysis was employed. MS demonstrated that hT40 and hT39 share several of the same Glc S/T residues, including T175, T181, S400, S413/T414/S416, S422, T427, and S433 (Fig. 1*B*). In addition, GlcNAc modifications on S184/S185 and S195/S198/S199 were detected only in Glc hT40, while modifications on T386 and S396 were detected only in Glc hT39 (Fig. 1*B*). In both tau isoforms, GlcNAc sites were concentrated within the proline-rich domain (PRD) and C terminus, while the MTBRs were not modified by O-GlcNAc. The O-GlcNAc modifications were detected by mass shifts and diagnostic ions in Glc hT40 and hT39 samples. The diagnostic ions observed with Glc tau proteins and mass spectra of the peptide spanning amino acids 402 to 429 from the Glc hT40 and hT39 protein preparations are shown in Figs. S1 and S2.

### O-GlcNAc tau does not alter microtubule polymerization *in vitro*

**Figure 2:**
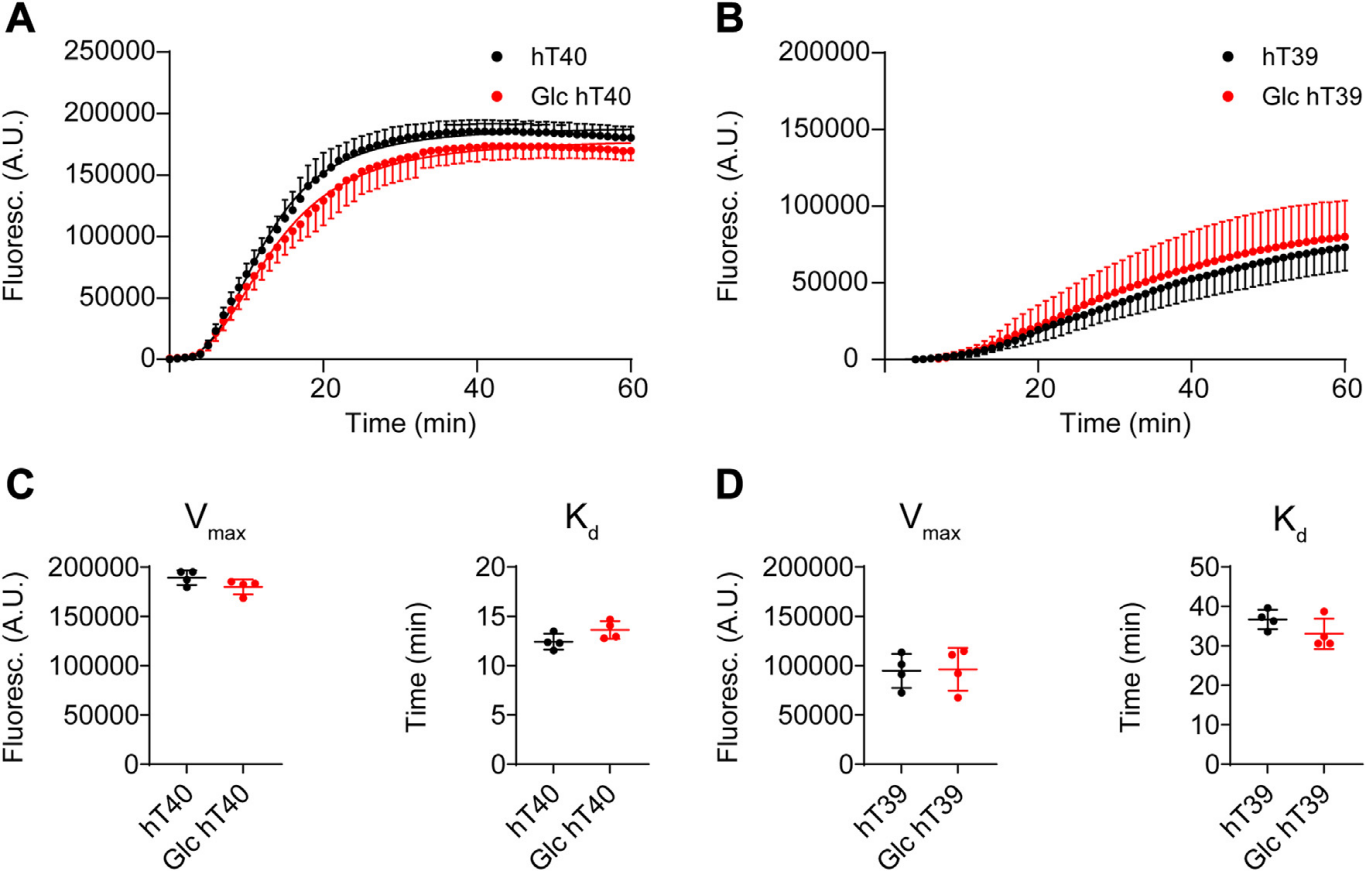
**O-GlcNAc modification of tau does not alter tubulin polymerization *in vitro*.***A*, curve fit of fluorescence signal corresponding to microtubule polymerization in the presence of either unmodified hT40 (*black*) or O-linked-N-acetyl β-d-N-glucosamine modified (Glc) hT40 (*red*) over 60 min. *B*, curve fit of fluorescence signal corresponding to microtubule polymerization in the presence of either unmodified hT39 (*black*) or Glc hT39 (*red*) over 60 min. *C*, polymerization rate (K_d_) of microtubule polymerization for unmodified hT40 and Glc hT40 (*left*). Steady state (V_max_) of microtubule polymerization for unmodified hT40 and Glc hT40 proteins (*right*). *D*, K_d_ of microtubule polymerization for unmodified hT39 and Glc hT39 (*left*). V_max_ of microtubule polymerization for unmodified hT39 and Glc hT39 proteins (*right*). Data represent the mean ± SD of four independent replicates.

Tubulin polymerization assays were used to determine how O-GlcNAc modification affects the ability of tau to modulate microtubule polymerization kinetics *in vitro* (Fig. 2, *A* and *B*). Glc hT40 did not change the polymerization rate (K_d_; t = 1.978, *p* > 0.05) or the steady-state polymerization (V_max_; t = 1.745, *p* > 0.05) of microtubules when compared to unmodified hT40 (Fig. 2*C*). Similarly, Glc hT39 did not alter the polymerization rate (K_d_; t = 1.583, *p* > 0.05) or the steady-state polymerization (V_max_; t = 0.1206, *p* > 0.05) of microtubules relative to unmodified hT39 (Fig. 2*D*).

### O-GlcNAc modification differentially affects binding of tau to microtubules *in vitro*

**Figure 3:**
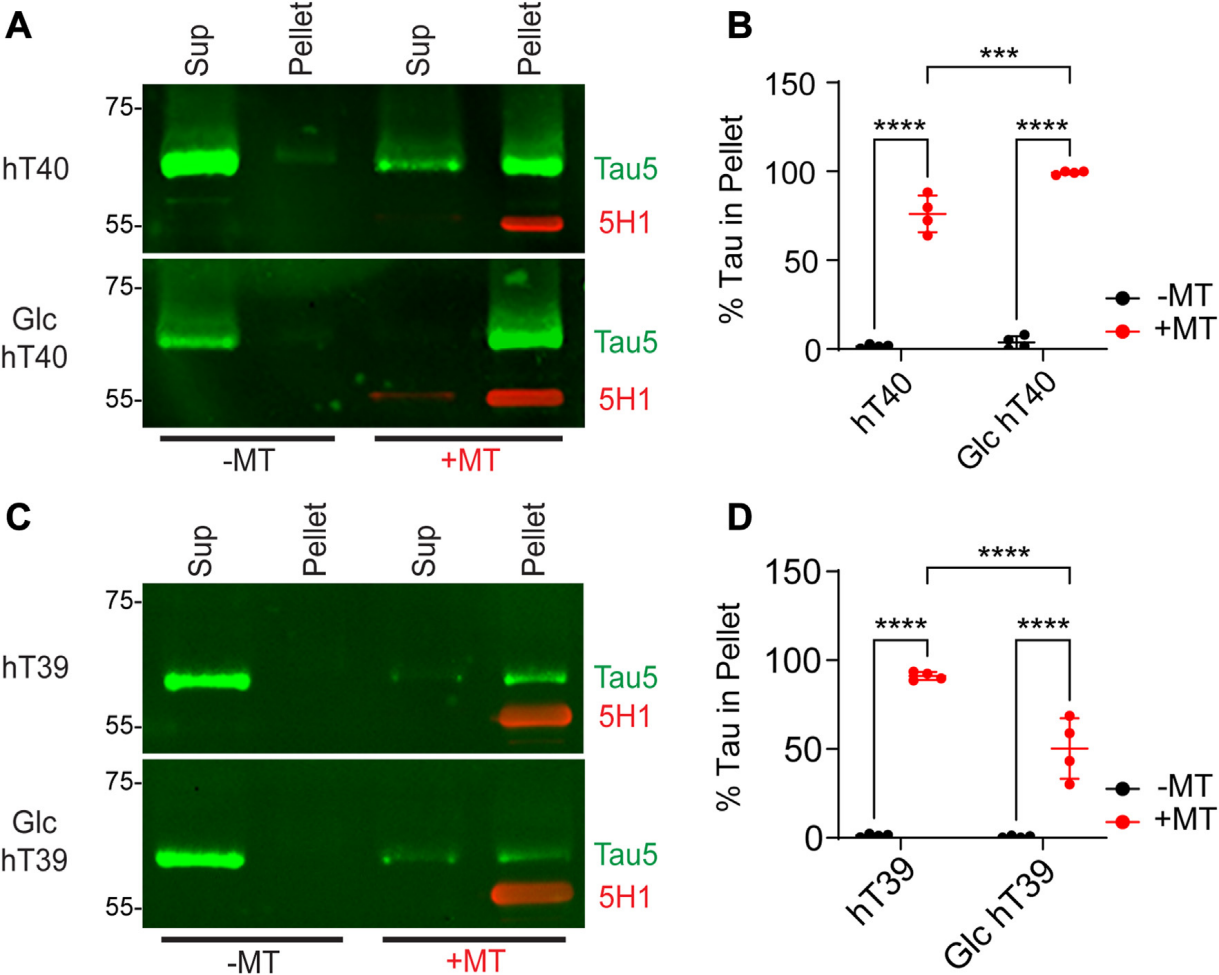
**O-GlcNAc modification alters binding of tau to microtubules *in vitro*.***A*, Western blot of microtubule-binding assay supernatant (Sup) and pellet samples for unmodified and O-linked-N-acetyl β-d-N-glucosamine modified (Glc) hT40 proteins using Tau5 (*green*) and 5H1 (tubulin, *red*) antibodies. *B*, quantification of the fraction of tau detected in the pellet of polymerized microtubules (MTs) for unmodified and Glc hT40. *C*, Western blot of microtubule-binding assay for unmodified and Glc hT39 proteins using Tau5 (*green*) and 5H1 (tubulin, *red*) antibodies. *D*, quantification of the fraction of tau detected in the pellet of polymerized microtubules for unmodified and Glc hT39. Data represent the mean ± SD of four independent replicates. ∗*p* ≤ 0.05; ∗∗*p* ≤ 0.01; ∗∗∗*p* ≤ 0.001; and ∗∗∗∗*p* ≤ 0.0001.

Microtubule-binding assays were used to determine how O-GlcNAc modification affects tau’s ability to bind preformed microtubules. Experiments were conducted using either the hT40 or hT39 tau isoforms independently (Fig. 3, *A* and *C*). Two-way ANOVA analysis revealed a statistically significant interaction between the PTM status of proteins and the presence of microtubules [*F*_(1, 12)_ = 14.59, *p* < 0.05]. Post hoc analysis showed a statistically significant shift of unmodified and Glc hT40 into the pellet fraction with preformed microtubules (t = 19.10, *p* < 0.05 for hT40; t = 24.50, *p* < 0.05 for Glc hT40). Moreover, there was a significant 30% increase of Glc hT40 in the pelleted microtubule fraction relative to unmodified hT40 (t = 5.953, *p* < 0.05; Fig. 3*B*).

In the case of hT39 isoform, two-way ANOVA showed a statistically significant interaction between the PTM status of proteins and the presence of microtubules [*F*_(1, 12)_ = 21.85, *p* < 0.05]. Post hoc analysis demonstrated a statistically significant shift of unmodified and Glc hT39 into the pellet fraction with preformed microtubules (t = 14.77, *p* < 0.05 for hT39; t = 8.164, *p* < 0.05 for Glc hT39). Contrary to Glc hT40, Glc hT39 in the pelleted microtubule fraction was significantly lower than unmodified hT39 by 45% (t = 6.731, *p* < 0.05; Fig. 3*D*).

### O-GlcNAc modification of tau alters its aggregation kinetics *in vitro*

**Figure 4:**
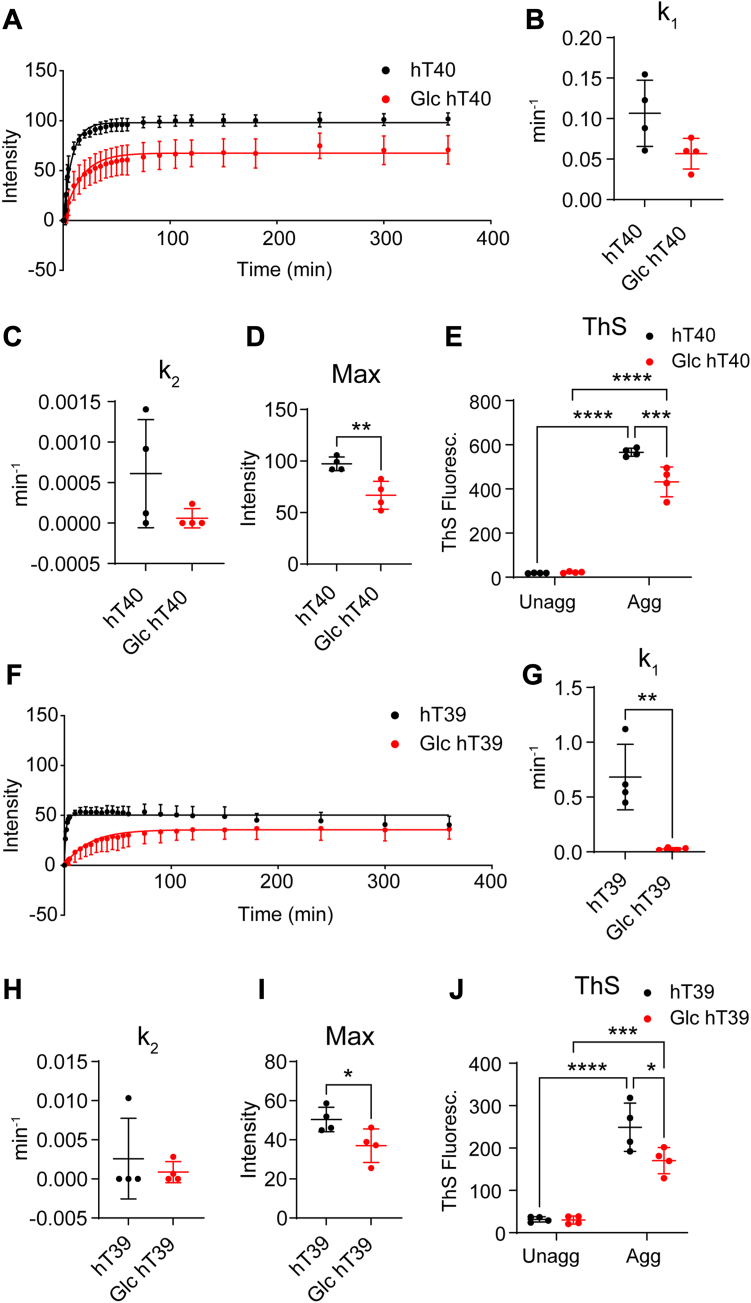
**O-GlcNAc modification of tau decreases the extent of tau polymerization and β-sheet**–**containing aggregates *in vitro*.***A*, laser light scattering (LLS) assay of unmodified hT40 and O-GlcNAc, O-linked-N-acetyl β-d-N-glucosamine modified (Glc) hT40. *B*, nucleation rate constant (k_1_) for aggregation of unmodified hT40 and Glc hT40 proteins. *C*, elongation rate constant (k_2_) for aggregation of unmodified hT40 and Glc hT40. *D*, maximum light scattered (Max) with polymerization of unmodified hT40 and Glc hT40. *E*, thioflavin S (ThS) fluorescence for unaggregated (Unagg) and aggregated (Agg) unmodified hT40 and Glc hT40. *F*, LLS of unmodified hT39 and Glc hT39. *G*, k_1_ for aggregation of unmodified hT39 and Glc hT39 proteins. *H*, k_2_ for aggregation of unmodified hT39 and Glc hT39. *I*, max polymerization for unmodified hT39 and Glc hT39. *J*, ThS fluorescence for Unagg and Agg unmodified hT39 and Glc hT39. Data represent the mean ± SD of four independent replicates. ∗*p* ≤ 0.05; ∗∗*p* ≤ 0.01; ∗∗∗*p* ≤ 0.001; and ∗∗∗∗*p* ≤ 0.0001.

Right-angle laser light scattering (LLS) assays were used to determine the impact of O-GlcNAc modification on the kinetics of tau aggregation *in vitro* (Fig. 4, *A* and *F*). We observed 32% reduction of the maximum light scattering with Glc hT40 compared to the unmodified hT40 (t = 4.042, *p* < 0.05; Fig. 4*D*). However, O-GlcNAc modification did not affect the rate constants of nucleation (t = 2.212, *p* > 0.05; Fig. 4*B*) or elongation (Mann–Whitney *U* = 4, *p* > 0.05; Fig. 4*C*) of hT40 protein. O-GlcNAc modification decreased the maximum light scattering of hT39 proteins by 27% (t = 2.523, *p* < 0.05; Fig. 4*I*) and the rate constant of nucleation by 65% (Mann–Whitney *U* = 0, *p* < 0.05; Fig. 4*G*). However, O-GlcNAc modification did not affect the rate of elongation (Mann–Whitney *U* = 7, *p* > 0.05; Fig. 4*H*).

### O-GlcNAc modification of tau reduces formation of **β**-sheet–containing aggregates *in vitro*

Thioflavin S (ThS) assays were performed at the end of aggregation reactions to determine the extent of β-sheet containing aggregate formation *in vitro*. Two-way ANOVA revealed a statistically significant interaction between the PTM and aggregation status of proteins [*F*_(1, 12)_ = 15.62, *p* < 0.05 for hT40 proteins; *F*_(1, 12)_ = 5.548, *p* < 0.05 for hT39 proteins; Fig. 4, *E* and *J*]. Upon aggregating hT40 and hT39 proteins, there was a significant increase in the ThS signal compared to their respective unaggregated samples regardless of the O-GlcNAc modification (t = 22.19, *p* < 0.05 for unmodified hT40; t = 16.6, *p* < 0.05 for Glc hT40; t = 9.383, *p* < 0.05 for unmodified hT39; t = 6.052, *p* < 0.05 for Glc hT39; Fig. 4, *E* and *J*). Moreover, the aggregates of Glc hT40 and hT39 showed significantly lower ThS fluorescence than their respective unmodified tau aggregates (24% reduction, t = 5.448, *p* < 0.05 for hT40 proteins; 32% reduction, t = 3.394, *p* < 0.05 for hT39 proteins; Fig. 4, *E* and *J*).

### O-GlcNAc modification differentially alters the size of tau aggregates *in vitro*

**Figure 5:**
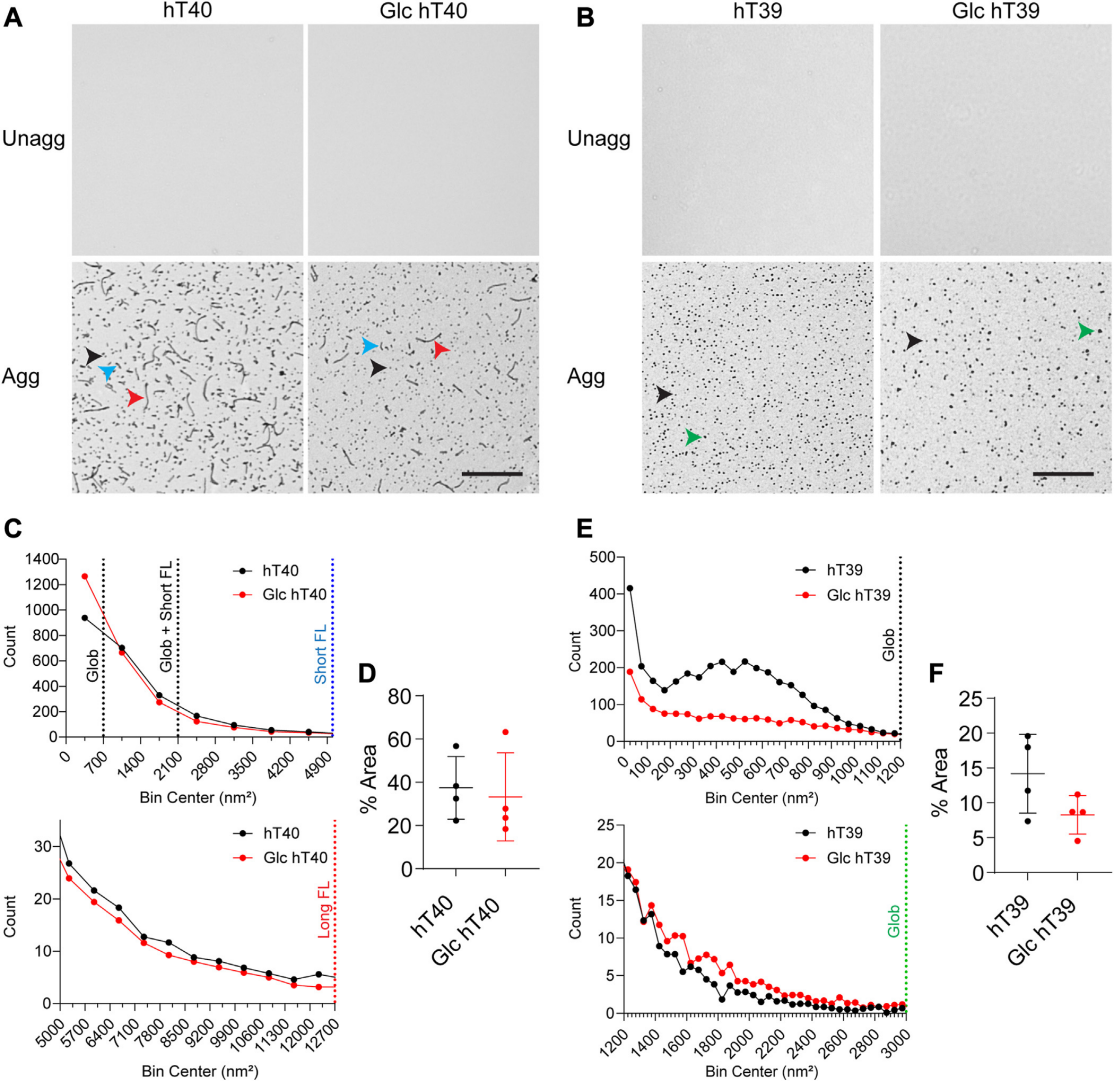
**O-GlcNAc modification alters the size distribution of tau aggregates *in vitro*.***A*, electron micrographs of unaggregated (Unagg) and aggregated (Agg) unmodified hT40 or O-GlcNAc, O-linked-N-acetyl β-d-N-glucosamine modified (Glc) hT40 proteins. The scale bar represents 800 nm. Globular aggregates <700 nm^2^ highlighted with *black arrowheads*; short (2100–5000 nm^2^) and long (>5000 nm^2^) filaments highlighted with *blue and red arrowheads*, respectively. *B*, electron micrographs of Unagg and Agg unmodified hT39 or Glc hT39 proteins. The scale bar represents 800 nm. Globular aggregates <1200 nm^2^ highlighted with *black arrowheads*; globular aggregates >1200 nm^2^ highlighted with *green arrowhead*. *C*, size distribution of aggregates formed by unmodified and Glc hT40 proteins. *D*, total mass of aggregates observed with unmodified and Glc hT40 expressed as percentage of field area (% area). *E*, size distribution of aggregates formed by unmodified and Glc hT39 proteins. *F*, total mass of aggregates observed with unmodified and Glc hT39 expressed as percentage of field area. Data represent the mean ± SD of four independent replicates. ∗*p* ≤ 0.05; ∗∗*p* ≤ 0.01; ∗∗∗*p* ≤ 0.001; and ∗∗∗∗*p* ≤ 0.0001.

Upon investigating the unaggregated and aggregated hT40 samples using transmission electron microscopy (TEM) (Fig. 5*A*), there was no significant change in the aggregated mass of Glc hT40 compared to unmodified hT40 (Fig. 5*D*; t = 0.3362, *p* > 0.05). Of note, the number of globular aggregates <700 nm^2^ was increased with Glc hT40 (Fig. 5*C*). On the other hand, the number of long filamentous aggregates (>5000 nm^2^) decreased with Glc hT40 compared to unmodified hT40 (Fig. 5*C*).

There was no statistically significant difference in the aggregated mass between unmodified and Glc hT39 (t = 1.875, *p* > 0.05; Fig. 5*F*). The size distribution of globular aggregates formed by Glc hT39 showed a decrease in the number of globular aggregates <1200 nm^2^ relative to the unmodified hT39 (Fig. 5*E*). Conversely, the number of globular aggregates >1200 nm^2^ was higher with Glc hT39 relative to unmodified hT39 (Fig. 5*E*).

### O-GlcNAc modification differentially alters the formation of stable tau multimers *in vitro*

**Figure 6:**
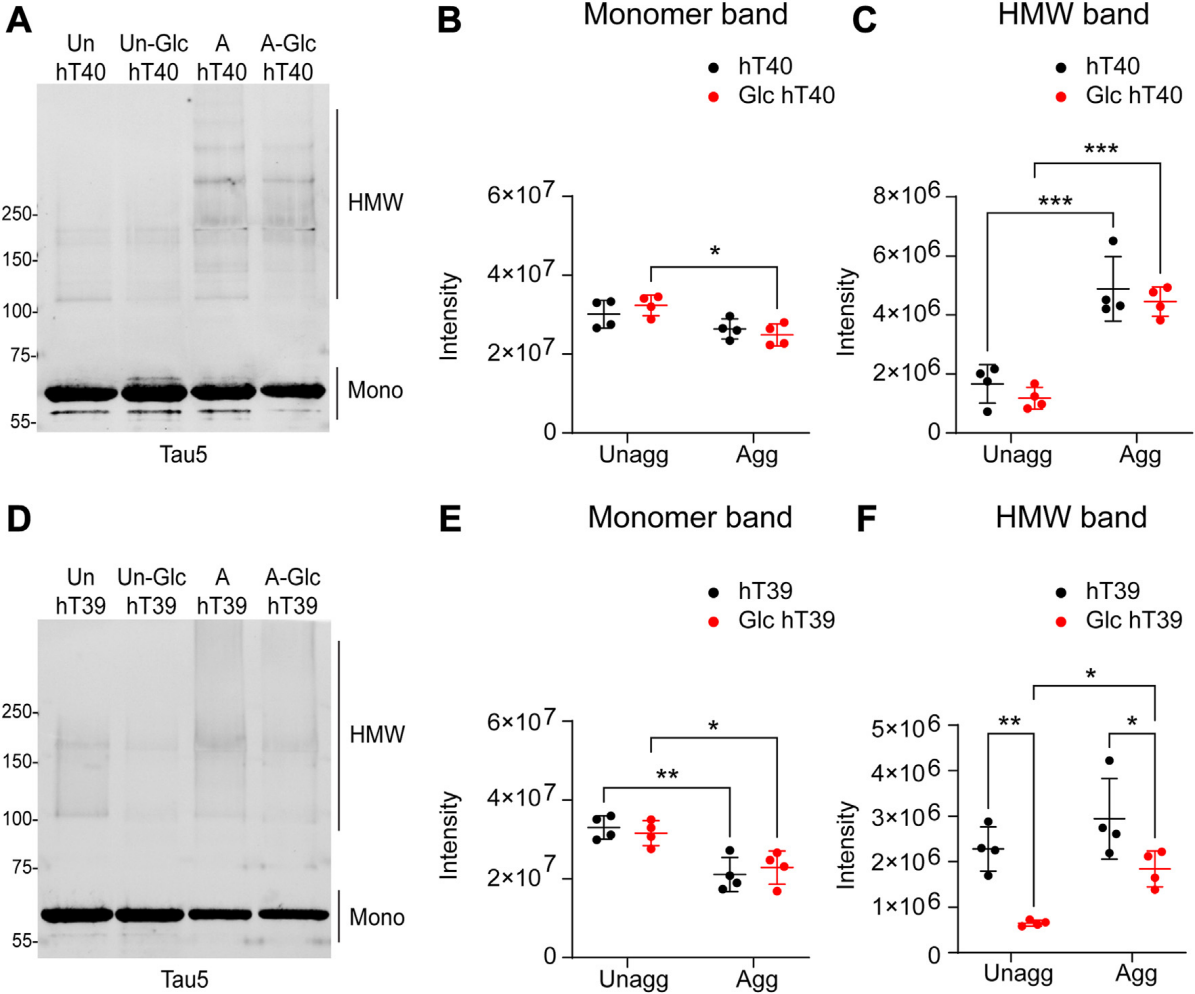
**O-GlcNAc modification of tau alters the formation of stable multimers.***A*, Western blot of unaggregated (Unagg) and aggregated (Agg) unmodified hT40 or O-GlcNAc, O-linked-N-acetyl β-d-N-glucosamine modified (Glc) hT40 samples probed with Tau5 antibody. *B*, quantification of monomeric (Mono) tau bands in hT40 samples. *C*, quantification of high molecular weight (HMW) tau bands in hT40 samples. *D*, Western blot of Unagg and Agg unmodified hT39 or Glc hT39 samples probed with Tau5 antibody. *E*, quantification of monomeric tau bands in hT39 samples. *F*, quantification of HMW tau bands in hT39 samples. Data represent the mean ± SD of four independent replicates. ∗*p* ≤ 0.05; ∗∗*p* ≤ 0.01; ∗∗∗*p* ≤ 0.001; and ∗∗∗∗*p* ≤ 0.0001.

Tau aggregation leads to the formation of heat-, SDS-, and reducing condition-stable multimers. Here, SDS-PAGE and Western blotting were employed to assess the extent to which O-GlcNAc modification of tau affects the formation of these stable multimers (Fig. 6, *A* and *B*). Two-way ANOVA showed a significant main effect of aggregation on the levels of stable multimers for hT40 proteins [Fig. 6*C*; *F*_(1, 12)_ = 83.99, *p* < 0.05]. A significant increase in the stable multimers upon aggregating hT40 proteins regardless of the O-GlcNAc modification was observed (3-fold increase, t = 6.431, *p* < 0.05 for unmodified hT40; 3.8-fold increase, t = 6.530, *p* < 0.05 for Glc hT40). Furthermore, two-way ANOVA revealed a significant main effect of aggregation on the levels of monomeric tau [Fig. 6*B*; *F*_(1, 12)_ = 14.98, *p* < 0.05]. The intensity of monomeric tau band was reduced by 23% in aggregated Glc hT40 when compared to unaggregated Glc hT40 (t = 3.657, *p* < 0.05). No difference was observed in the monomeric tau signal upon aggregating the unmodified hT40 (t = 1.816, *p* > 0.05).

We also assessed the extent to which O-GlcNAc modification affects the monomeric and stable multimers bands in hT39 proteins. Two-way ANOVA demonstrated a significant main effect of aggregation on the levels of monomeric tau in hT39 proteins (*F*_(1, 12)_ = 30.81, *p* < 0.05; Fig. 6*E*). There was a statistically significant reduction in monomeric tau signal upon aggregation regardless of the O-GlcNAc modification status (36% decrease, t = 5.536, *p* < 0.05 for unmodified hT39; 27.5% decrease, t = 3.314, *p* < 0.05 for Glc hT39). Furthermore, two-way ANOVA indicated a significant main effect of both aggregation and PTM status on the stable tau multimers (*F*_(1, 12)_ = 11.67, *p* < 0.05 for aggregation; *F*_(1, 12)_ = 25.32, *p* < 0.05 for PTM; Fig. 6*F*). Despite the increase in stable multimers upon aggregating Glc hT39 (t = 3.107, *p* < 0.05), *post hoc* analysis confirmed that stable multimers were lower with Glc hT39 proteins in the unaggregated and aggregated samples (72% decrease, t = 4.250, *p* < 0.05 for unaggregated samples; 37% decrease, t = 5.505, *p* < 0.05 for aggregated samples).

### O-GlcNAc modification differentially alters the formation of pathological tau conformations *in vitro*

**Figure 7:**
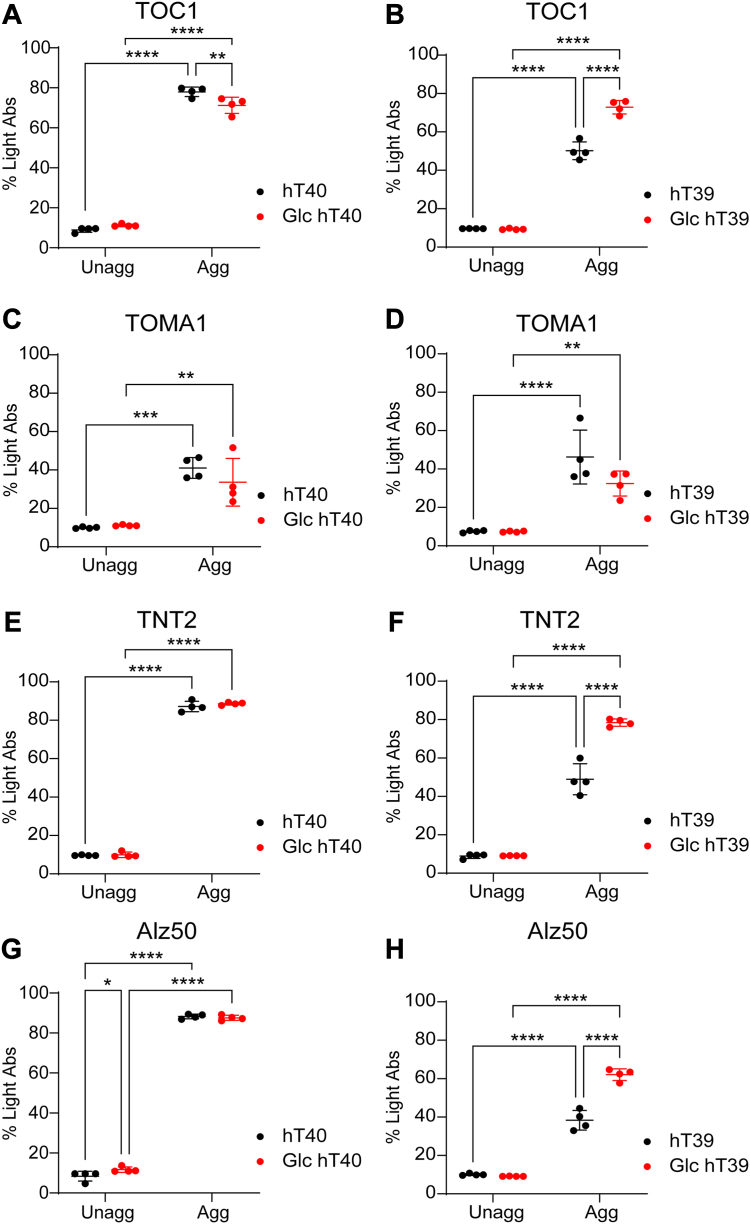
**O-GlcNAc modification of tau alters the formation of pathological conformations upon aggregation *in vitro***. *A* and *B*, sandwich ELISA assay measuring oligomeric tau in unaggregated (Unagg) and aggregated (Agg) unmodified and O-linked-N-acetyl β-d-N-glucosamine (Glc)-modified hT40 (*A*) and hT39 (*B*) proteins using TOC1 antibody for capture and R1 antibody for detection. *C* and *D*, sandwich ELISA assay measuring oligomeric tau in unaggregated and aggregated hT40 (*C*) and hT39 (*D*) proteins using TOMA1 antibody for capture and R1 antibody for detection. *E* and *F*, sandwich ELISA assay measuring PAD exposure in unaggregated and aggregated hT40 (*E*) and hT39 (*F*) proteins using TNT2 antibody for capture and R1 antibody for detection. *G* and *H*, sandwich ELISA assay measuring misfolded tau conformation in unaggregated and aggregated hT40 (*G*) and hT39 (*H*) proteins using Alz50 antibody for capture and R1 antibody for detection. Data represent the mean ± SD of four independent replicates. ∗*p* ≤ 0.05; ∗∗*p* ≤ 0.01; ∗∗∗*p* ≤ 0.001; and ∗∗∗∗*p* ≤ 0.0001. PAD, phosphatase-activating domain.

Several antibodies are available to detect oligomeric tau species, which are linked to toxicity and neurodegeneration (6, 36, 44, 64). Oligomeric tau species were quantified in unaggregated and aggregated tau samples using sandwich ELISAs with two different oligomeric tau antibodies (*i*.*e*., TOC1 and TOMA1). Two-way ANOVA demonstrated a significant interaction between the PTM and aggregation factors on the levels of TOC1-positive oligomeric tau in hT40 samples (*F*_(1, 12)_ = 13.65, *p* < 0.05). Post hoc analysis showed an increase in TOC1-positive oligomeric tau in the aggregated samples of unmodified and Glc hT40 relative to their respective unaggregated tau proteins (t = 40.09, *p* < 0.05 for unmodified hT40; t = 34.86, *p* < 0.05 for Glc hT40; Fig. 7*A*). In addition, aggregated Glc hT40 showed a small (8%) yet significant reduction in TOC1 signal relative to the aggregated unmodified hT40 samples (t = 3.916, *p* < 0.05). TOC1 sandwich ELISA assays with the hT39 protein samples [two-way ANOVA; Interaction: *F*_(1, 12)_ = 62.15, *p* < 0.05] revealed increased TOC1-positive oligomeric tau in the aggregated samples of unmodified and Glc hT39 proteins relative to their respective unaggregated samples (t = 19.66, *p* < 0.05 for unmodified hT39; t = 30.81, *p* < 0.05 for Glc hT39; Fig. 7*B*). In contrast to Glc hT40, aggregated Glc hT39 samples had 45% higher TOC1-positive oligomeric tau relative to the aggregated samples of unmodified hT39 (t = 11.02, *p* < 0.05; Fig. 7*B*).

Next, we assessed the levels of TOMA1-positive oligomeric tau species in the unmodified and Glc tau samples. Aggregation of unmodified and Glc hT40 elicited an increase in the TOMA1 signal when compared to the unaggregated samples (aggregation factor: *F*_(1, 12)_ = 62.43, *p* < 0.05; t = 6.484, *p* < 0.05 for unmodified hT40; t = 4.691, *p* < 0.05 for Glc hT40; Fig. 7*C*). The same finding was also observed upon the aggregation of unmodified and Glc hT39 proteins, where the TOMA1 signal was increased compared to the unaggregated hT39 tau samples (aggregation factor: *F*_(1, 12)_ = 67.75, *p* < 0.05; t = 19.66, *p* < 0.05 for unmodified hT39; t = 30.81, *p* < 0.05 for Glc hT39; Fig. 7*D*). Unlike the TOC1 results, no differences were observed between unmodified and Glc hT40 and hT39 proteins in unaggregated or aggregated samples.

Tau is known to adopt pathological conformations associated with modifications of monomeric tau and following multimerization (6, 51, 53, 55). These conformations include exposure of the PAD in tau that spans amino acids 2 to 18 and whose abnormal exposure is linked to dysfunction of axonal transport (51, 54, 65). Another misfolded conformation involves the N terminus of tau coming into close proximity to the MTBR region of tau that is thought to occur early in disease and precede tau aggregation (58, 59, 66).

The extent to which O-GlcNAc modification can influence the adoption of pathological conformations was assessed using sandwich ELISA with the TNT2 antibody for PAD exposure and the Alz50 antibody for the misfolding. Regardless of O-GlcNAc modification, aggregated hT40 samples showed higher TNT2 signal than the unaggregated samples (aggregation factor: *F*_(1, 12)_ = 10,007, *p* < 0.05; t = 70.19, *p* < 0.05 for unmodified hT40; t = 71.28, *p* < 0.05 for Glc hT40; Fig. 7*E*). On the other hand, two-way ANOVA demonstrated a significant interaction between the PTM and aggregation factors on the levels of TNT2 signal in hT39 samples (*F*_(1, 12)_ = 48.81, *p* < 0.05). The TNT2 signal was higher in the aggregated hT39 relative to the unaggregated samples regardless of their modification status (t = 13.48, *p* < 0.05 for unmodified hT39; t = 23.36, *p* < 0.05 for Glc hT39; Fig. 7*F*). No differences in TNT2 signal were detected among the unaggregated samples of unmodified and Glc hT39 proteins (t = 0.06469, *p* > 0.05). However, the TNT2 signal in the aggregated Glc hT39 samples was 60% higher than the aggregated samples of unmodified hT39 (t = 9.945, *p* < 0.05).

Two-way ANOVA demonstrated a significant interaction between the PTM and aggregation factors on the levels of Alz50 signal in both hT40 (*F*_(1, 12)_ = 6.067, *p* < 0.05) and hT39 samples (*F*_(1, 12)_ = 6.067, *p* < 0.05). Alz50-positive misfolded tau was higher in the aggregated hT40 samples relative to the unaggregated samples regardless of the O-GlcNAc modification (t = 68.42, *p* < 0.05 for unmodified hT40; t = 64.93, *p* < 0.05 for Glc hT40; Fig. 7*G*). Of note, Alz50-positive misfolded conformation was significantly increased by 40% in the unaggregated Glc hT40 relative to unmodified hT40 (t = 2.832, *p* < 0.05). However, no difference was observed in the Alz50 signal between the aggregated samples of unmodified and Glc hT40 proteins (t = 0.6514, *p* > 0.05). Regardless of the O-GlcNAcylation status of proteins, aggregated hT39 samples showed higher Alz50 signal than the unaggregated samples (t = 13.33, *p* < 0.05 for unmodified hT39; t = 24.95, *p* < 0.05 for Glc hT39; Fig. 7*H*). On the other hand, aggregated Glc hT39 showed higher Alz50-positive tau relative to unmodified hT39 aggregates (t = 11.19, *p* < 0.05).

For all unaggregated and aggregated hT40 and hT39 proteins, total tau levels were measured using Tau13 as capture antibody (Fig. S3). No differences were observed in total tau levels across the hT40 (two-way ANOVA; interaction: *F*_(1, 12)_ = 0.06549, *p* > 0.05; aggregation factor: *F*_(1, 12)_ = 1.092, *p* > 0.05; PTM factor: *F*_(1, 12)_ = 1.296, *p* > 0.05) or the hT39 samples (two-way ANOVA; interaction: *F*_(1, 12)_ = 0.6154, *p* > 0.05; aggregation factor: *F*_(1, 12)_ = 0.2425, *p* > 0.05; PTM factor: *F*_(1, 12)_ = 2.012, *p* > 0.05).

## Discussion

PTMs regulate several aspects of tau protein biology including aggregation, conformational changes, microtubule binding, degradation, and clearance (6, 10). Tau is subject to modification with O-GlcNAc as reported in studies utilizing recombinant proteins, cell lines, animal models, and AD brains (20, 21, 24, 27, 67, 68). O-GlcNAcylation of the longest tau isoform (2N4R isoform, hT40) competes with phosphorylation and inhibits formation of β-sheet containing tau aggregates (23, 25, 27). The previously observed effects of O-GlcNAcylation on tau biology are partially attributed to modifying S400 (24, 69). In this work, we show that tau is modified by O-GlcNAcylation on S400 in addition to several novel sites (*e*.*g*., T175, T181, S422, S427, and S433) (25, 67). Of note, O-GlcNAcylation sites are concentrated within the PRD and C-terminal domain of tau (6, 70).

As mentioned previously, we observed GlcNAc modification at sites not previously reported, such as T175, T181, S422, S427, and S433. Similarly, previous MS studies identified O-GlcNAc modification at sites that were not identifiable in our study such as S208, S238, and T231 (25, 67). This difference in detectable modification sites is not surprising given the challenging nature of detecting GlcNAc sites using MS (71). Furthermore, variations in recombinant protein preparation, bacterial strains used for cotransformation, digestion conditions, and MS detection protocols may explain differences in O-GlcNAc sites identified among different studies.

Several of the GlcNAc modification sites, including the novel sites identified in our study, are potentially important in the context of disease because they are phosphorylated in tauopathies. For example, phosphorylation at T175 and T181 in the PRD of tau increases significantly in multiple tauopathies, such as AD, PiD, and CBD (11, 20, 72, 73, 74, 75, 76, 77). On the C-terminus of tau, phosphorylation at S422 and the paired helical filament-1 epitope (S396/S404) is upregulated early in several tauopathies (11, 78, 79, 80, 81, 82, 83). It is intriguing that reduced O-GlcNAcylation of tau is coincident with increased phosphorylation at S422 and paired helical filament-1 (20). Therefore, the relationship between O-GlcNAcylation and phosphorylation of these sites warrants further investigation (27, 69).

The functional implications of O-GlcNAc modification of tau are not well studied; however, PTMs of tau are known to impact its ability to modulate microtubule dynamics and binding (84, 85, 86, 87, 88, 89, 90, 91). Our results show that the rate and extent of microtubule polymerization did not change in the presence of Glc tau proteins. In agreement, the Vocadlo group published similar results where Glc hT40 did not alter microtubule polymerization (24). On the other hand, binding to microtubules was reduced with Glc hT39 and increased with Glc hT40. These findings suggest that O-GlcNAcylation differentially regulates the binding of tau isoforms to microtubules.

The opposite effect on hT40 and hT39 is a novel finding, which may be explained by the additional hT39-specific modification of T386 and S396 in the fifth MTBR of tau (R′). Indeed, a recent study utilized NMR to elucidate the role of tau jaws in binding to microtubules (92, 93, 94). NMR spectra indicated that the R′ binds to taxol-stabilized microtubules with an affinity higher than the other MTBRs (70). In addition, this interface between microtubules and R′ region is sequestered away from water, likely by the dynamic second PRD (P2) and the first MTBR (R1) (70). The additional modifications at T386 and S396, along with S400, on R′ of hT39 may disrupt the sequestration of the R′ microtubule interface from water by modifying the net charge of R′. On the other hand, hT40 has less GlcNAc modification sites at the R′ region but relatively more abundant O-GlcNAc at S184/S185/S195 and S198/S199 of the P2 region. The heavier density of GlcNAc at the P2 region may enhance water sequestration of the R′ microtubule interface mediated by the dynamicity of P2 and R1 regions. Taken together, these findings suggest that O-GlcNAcylation may regulate microtubule binding of tau in an isoform-dependent manner.

O-GlcNAcylation of tau decreases the extent of tau aggregation (LLS) with a reduction in β-sheet–containing aggregates (ThS). In addition, TEM demonstrated that GlcNAc modification of hT40 reduces the formation of long filamentous tau aggregates. Corroborating our results, Yuzwa *et al.* previously reported reduced aggregation and β-sheet content of Glc full-length tau (24). On the other hand, the size of globular hT39 aggregates increases with O-GlcNAcylation, suggesting this modification has an impact on nonfilamentous tau multimerization. Additional evidence that O-GlcNAcylation differentially regulates the multimerization of tau isoforms was the direction of change in stable multimers formed upon aggregation (36, 95). Glc hT40 showed an increase in stable multimers upon aggregation comparable to that observed with unmodified hT40. Conversely, the stable multimers are reduced both in the unaggregated and aggregated conditions of Glc hT39 when compared to unmodified hT39. The slightly increased size of multimers formed by Glc hT39 along with their reduced stability under SDS and heat suggests that their conformation may be different from the multimers formed by the unmodified hT39.

PTMs are closely linked to processes associated with tau pathobiology, including the adoption of pathological conformations linked to tauopathies (6). PAD exposure is a pathological tau conformation that occurs early in AD and is linked to dysregulation of axonal transport (38, 46, 51, 52, 56). The resultant hyperactivation of the protein phosphatase 1-glycogen synthase kinase 3 β pathway mediates the PAD exposure-induced dysregulation of axonal transport (54, 65). In our experiments, we measured the extent of PAD exposure using the TNT2 antibody (38). We demonstrated that O-GlcNAc modification of hT39 enhances PAD exposure in aggregated conditions, and that the extent of PAD exposure was similar between hT40 and Glc hT40.

In addition, oligomerization of tau is an event detected in early Braak stages (starting from stage I) before the development of cognitive symptoms in AD patients (37, 96). Tau oligomers are linked to several disease mechanisms including axonal transport impairment (36, 43, 97), mitochondrial and synaptic dysfunction (98), reduced protein synthesis (99), inhibited long-term potentiation (100), and memory impairment (101). The process of tau oligomerization can be modulated by PTMs such as phosphorylation at the S199, S202, and T205 (61). In our experiments, we measured the abundance of oligomeric tau species using TOC1 (36, 64) and TOMA1 antibodies (44, 45). O-GlcNAc modification of hT39 increases the oligomeric tau species in aggregated samples, suggesting that this PTM may further exacerbate the dysfunctions associated with hT39 oligomers. On the other hand, O-GlcNAcylation of hT40 does not alter TOMA1-positive tau, while slightly reducing the TOC1-positive tau in aggregated hT40 samples. Thus, O-GlcNAcylation may again show isoform-dependent differences in the adoption of oligomeric tau species.

Early in disease, tau assumes a misfolded conformation in tauopathies that involves the N terminus of tau folding over its MTBR region and is thought to precede the formation of filamentous tau aggregates (59). This misfolded conformation is detected with the Alz50 and MC1 antibodies originally developed against paired helical filaments isolated from AD (58, 102). Nonetheless, these misfolded tau conformations are detectable in tauopathies other than AD, such as CBD, PiD, and PSP (11). Our results showed that GlcNAc modification of hT39 increases the Alz50 conformation in aggregated hT39 samples. This finding suggests that GlcNAc modification may increase the propensity of hT39 to adopt aggregation-prone conformations. In fact, our TEM data suggests that Glc hT39 forms globular aggregates that are slightly larger in size than those formed by the unmodified hT39 protein. O-GlcNAcylation of hT40 did not alter the extent of Alz50 conformation formed, suggesting isoform-dependent differences exist with this conformation as well.

Collectively, our findings add to the growing body of literature showing that enzymatic or nonenzymatic PTMs may differentially regulate the 4R and 3R tau isoforms (103, 104, 105, 106). For example, enzyme-mediated acetylation inhibits the aggregation of 2N4R tau isoform but increases the aggregation of the 0N3R isoform (105). In addition, nonenzymatic glycation enhances aggregation of the 2N4R tau isoform but inhibits aggregation of the 1N3R isoform (103).

The effects that O-GlcNAcylation has on the different tau isoforms highlights important considerations for testing OGA inhibitors in animal models and clinical trials. OGA inhibitors were tested for their utility in treating tauopathies using animal models that express only 4R human tau isoforms (28, 29, 30, 107). Animal studies suggested that OGA inhibitors are beneficial in reducing tangle pathology; hence, they were advanced in the clinical trial pipeline for AD (108, 109, 110). However, AD pathology is composed of both 4R and 3R proteins, and other tauopathies accumulate pathology mainly composed of only 3R tau (*e*.*g*., PiD) (1). It would be beneficial to test OGA inhibitors in mouse models that express 3R tau isoforms, such as mThy-1 3R Tau (111). Given the differential regulation of tau isoforms by O-GlcNAcylation, it is plausible that OGA inhibitors also differentially affect the tauopathy phenotype in mice expressing 4R *versus* 3R tau isoforms.

Multiple OGA inhibitors were advanced to phase 1 clinical trials and were well tolerated, safe, showed favorable pharmacokinetics, were brain permeable, and had high enzyme occupancy (112, 113, 114, 115, 116), https://www.alzforum.org/therapeutics/ly3372689, https://www.alzforum.org/therapeutics/asn90 (117). However, the recent PROSPECT-ALZ phase 2 clinical trial (NCT05063539) testing the OGA inhibitor, Ceperognastat (LY3372689), in early symptomatic AD individuals failed to meet primary and secondary clinical symptom improvement outcomes https://www.alzforum.org/news/conference-coverage/tau-modification-drugs-take-hit-negative-trial. The high-dose Ceperognastat treatment appeared to significantly worsen symptoms and elevate off-target adverse events; however, there was a reduction in hippocampal atrophy, less temporal lobe tau pathology (as measured by PET imaging with flortaucipir), and lower plasma pTau-217 levels. The mechanisms behind these discordant results remain unclear but may involve off-target effects of OGA inhibition in peripheral and/or brain tissues (118, 119, 120). In addition, GlcNAc modification can impact tau phosphorylation (20, 21, 25, 26), enabling OGA inhibitors to introduce other changes in the PTM profile of tau. Perhaps an intervention approach with OGA inhibitors may require cotreatments targeting other PTMs that contribute to tau pathology, such as phosphorylation. Considering the complexity of tau PTMs, a deeper understanding of the site-specific effects of GlcNAc on tau is critical. If our findings are translatable, blocking the removal of GlcNAc modifications *via* OGA inhibitors could enhance some pathogenic conformations of 3R tau isoforms (*i.e.*, TOC1+ oligomers and PAD exposure). Notably, both tau isoforms showed evidence of reduced β-sheet–containing aggregates (*i*.*e*., ThS-positive), which are the targets of current tau PET tracers (121). Thus, while OGA inhibitor therapeutics hold promise and are still pursued clinically, a cautious approach is warranted. Further work is essential to enhance brain-specific targeting, explore combinatorial interventions, and elucidate how O-GlcNAc modification impacts PTMs at specific tau sites.

In summary, we report novel O-GlcNAc modification sites on the hT40 and hT39 isoforms of tau. O-GlcNAcylation affects several aspects of the pathobiology of tau in an isoform-dependent manner. In the case of hT40, O-GlcNAcylation increases microtubule binding and decreases the filamentous and β-sheet–containing aggregates without altering the formation of pathological tau conformations (*e*.*g*., oligomerization and PAD exposure). In the case of hT39, O-GlcNAcylation decreases microtubule binding, decreases β-sheet–containing aggregates, and markedly enhances the formation of pathological tau conformations (*e.g.*, oligomerization and PAD exposure). Further studies in animal models and results from clinical trials will provide further insights into the role played by O-GlcNAcylation in contributing to or mitigating tauopathies (108, 109, 110).

## Experimental procedures

### Cloning of O-GlcNAc transferase enzyme

The coding sequence (CDS) of human O-GlcNAc transferase (OGT) was amplified by PCR from the OGT ORF vector (Applied Biological Materials Inc., # 32487011) using the Platinum PCR Supermix High Fidelity (Invitrogen, #12532016). Forward and reverse primers were designed to amplify OGT CDS while also introducing NdeI and XhoI restriction sites to the forward and reverse primers, respectively.

Forward primer: CATATGATGGCGTCTTCCGTGGG (NdeI site underlined)

Reverse primer: CTCGAGTTATGCTGACTCAGTGACTTCAA (XhoI site underlined)

The PCR product was subjected to A tailing using native Taq polymerase (Invitrogen, #18038-042) followed by ligation into the pCR2.1-TOPO backbone using the TOPO TA cloning (Invitrogen, #450640). The sequence of OGT CDS was confirmed using Sanger sequencing. OGT CDS was then digested with NdeI (Thermo, #FD0583) and XhoI (Thermo, #FD0694), ran on 1% agarose gel (Bio-Rad, #1613101), and the OGT CDS band was extracted using GeneJET Gel Extraction Kit (Thermo, #K0691). In parallel, the pHis-hTG2 plasmid (Addgene, #100719) was digested with the same enzymes, ran on agarose gel, and the vector backbone band extracted. The vector and OGT CDS DNA were ligated using the T4 enzyme (Thermo, #EL0011). The ligation product was used to transform TOP10 Chemically Competent cells (Thermo, #C404010) before plating the competent cells in agar plates (Fischer Bioreagents, #BP1423-500) supplemented with kanamycin at 50 mg/ml (Sigma, #K1876). Colonies were picked up from the agar plate, followed by shaking (250 RPM) in LB (Fischer Bioreagents, #BP1426-2) at 37 °C overnight. On the following day, the DNA plasmid was extracted from bacterial cultures using QIAprep Spin Miniprep Kit (Qiagen, #27104). The OGT CDS was verified with diagnostic digestion and Sanger sequencing.

### Preparation of recombinant unmodified and Glc tau proteins

Recombinant tau proteins were prepared by cotransforming BL21 bacteria (NEB, #C2527H) with two plasmids: a plasmid-expressing tau under the T7 promoter as described previously (122) and the pHis-OGT plasmid. The purification procedure for Glc tau proteins was performed using 2 L terrific broth (TB) cultures grown in the presence of ampicillin (50 μg/ml) and kanamycin (25 μg/ml) as selection markers. Moreover, TB was supplemented with GlcNAc (2 mM; Sigma, #U4375) and PUGNAc (10 μM; Sigma, #A7229) to enrich GlcNAc and inhibit the activity of OGA enzyme, respectively. Unmodified tau proteins were grown in the same way with the exception that kanamycin, GlcNAc, and PUGNAc were excluded from TB. Bacterial pellets were lysed using 0.5 M NaCl, 10 mM Tris, and 5 mM imidazole, pH 8 in the presence of protease inhibitors (as described previously in (122)) and PUGNAc (10 μM) at weight: volume ratio of 1:5. PUGNAc was not included in lysing the bacterial pellets for unmodified tau proteins. The bacterial lysate was subjected to centrifugation at 107,377 relative centrifugal force (RCF) for 45 min at 4 °C using a Type 70 Ti rotor (Beckman Coulter, #337922). Supernatant was collected, then the residual bacterial pellet was further lysed in radioimmunoprecipitation assay (RIPA) buffer (10 ml; Cell Signaling Technologies, #9806) supplemented with the same inhibitors by sonicating for 4 times, 30 s each. Another centrifugation step was performed to collect the supernatant extracted with the RIPA buffer, followed by pooling the two supernatants (lysis buffer and RIPA buffer) together for further purification. The rest of the purification procedure was performed as described previously (122). Briefly, three stages of fast protein liquid chromatography were performed: heavy metal affinity chromatography using a 5 ml HiTrap Talon crude column (Cytiva, #28953767); size-exclusion chromatography using HiPrep 16/60 Sephacryl S-500 HR (Cytiva, #28935606); anion exchange chromatography using 5 ml HiTrap Q HP (Cytiva, #17115401). The elution fractions containing the highly purified monomeric tau were concentrated to 2 to 4 mg/ml and supplemented with 1 mM DTT. The final unmodified and Glc tau proteins were aliquoted and frozen at −80 °C. The final concentration of recombinant tau proteins was determined using the SDS-Lowry method as described previously (122).

### Western blot validation of recombinant tau proteins

To confirm the O-GlcNAcylation status of recombinant tau proteins, proteins were loaded on 4 to 20% Novex tris-glycine gels (Invitrogen, #WXP42020BOX) and run at 225 V for 45 min, followed by transfer on nitrocellulose membrane using the Bio-Rad wet transfer system at 40 mAmp for 50 min. The membrane was then blocked with 2% nonfat dry milk (NFDM) in 1X tris buffer saline (TBS) for 1 h at room temperature (RT), followed by incubation in primary antibodies overnight at 4 °C. Primary antibodies used were Tau5 (Nicholas M. Kanaan at Michigan State University, RRID: AB_2721194) (123, 124) at 1:100,000 in 2% NFDM and Anti-Tau (GlcNAc Ser400) (Anaspec, #AS-55945) at 1:100 in 2% NFDM. The following day, the blot was washed three times (5 min each) with 1X TBS supplemented with 0.1% Tween 20. Then, secondary antibodies in 2% NFDM were added for 1 h at RT. Secondary antibodies used were peroxidase-conjugated goat anti-mouse IgG1 (Thermo Fisher Scientific, #PA1-74421, RRID: AB_10988195) at 1:5000 and peroxidase-conjugated goat anti-rabbit IgG (Vector Laboratories, #PI-1000-1, RRID: AB_2916034) at 1:2500. The blots were washed three times (5 min each) with TBS supplemented with 0.1% Tween 20, followed by the peroxidase development reaction using the Clarity Max Western ECL Substrate kit (Bio-Rad, #1705062S). ChemiDoc MP Imaging System and Image Lab 6.0.1 (https://www.bio-rad.com) were used to visualize the chemiluminescence signal verifying the O-GlcNAc tau modification status.

### Recombinant tau protein preparation for tandem MS

Unmodified and Glc tau proteins were digested using a combination of Asp-N (Promega, #V1621) and rLys-C (Promega, #V167A). First, each recombinant tau protein sample (10 μg, n = 1) was subjected to five rounds of buffer exchange with 25 mM ammonium bicarbonate (AmBic) pH 8 using 0.5 ml Amicon filter with 3K molecular weight cutoff (15,000 RCF for 10 min; Millipore, #UFC500396). Then, recombinant tau proteins were recovered from the filter by centrifugation at 15,000 RCF for 2 min and vacuum dried using Vacufuge. The dried pellets of recombinant tau proteins were reconstituted in 50 μl of digestion buffer (12.5 mM AmBic pH 8 + acetonitrile 50%) and incubated at 37 °C for 16 to 18 h with Asp-N (150 ng of enzyme). The following day, digested protein samples were subjected to vacuum drying and stored at 4 °C until the second digestion was initiated. Lys-C (500 ng of enzyme) was added and incubated at 37 °C for 16 to 18 h. The following day, digested protein samples were subjected to vacuum drying and stored at −20 °C until running on the MS.

### Tandem MS of recombinant tau proteins

We utilized an approach like that described by Yang *et al.* (125). MS analysis was performed twice: initially for method development of recombinant Glc tau and subsequently to validate the final protein preparations used for experiments. The Vanquish Neo nanoHPLC system interfaced to a Thermo Scientific Orbitrap Eclipse MS (Thermo Fisher Scientific) was used for analysis. For each sample, 1 μg was injected and desalted with an Acclaim PepMap C18 Nano trap column (3 μm, 100 Å, 75 μm × 2 cm) in 100% buffer A (0.1% formic acid in HPLC water) at 3 μl/min for 5 min. Samples were separated in a linear gradient of 5 to 35% buffer B (80% acetonitrile and 0.1% formic acid) over 105 min and washing at 90% buffer B for 12 min using an Easy Spray PepMap RSLC C18 nano column (2 μm, 100 Å, 75 μm × 250 mm). Before each injection, the column was equilibrated at 1.0% buffer B for 5 min. Mass spectra were collected using data-dependent MS analysis with a duty cycle of 2 s. To collect precursor masses, orbitrap [resolution (R) of 120,000 at 200 *m/z*] with internal calibration was used. For precursors carrying charges between 2 and 8 and with intensities over 5 × 10^4^ at R = 30,000, stepped higher-energy collisional dissociation spectra at energies of 15, 25, and 35% were acquired with dynamic exclusion of 15 s. The fragments are monitored for GlcNAc oxonium ions at *m/z* of 138.0545, 204.0867, 366.1396, 126.005, 144.0655, 168.0654, 186.076, 274.0921, and 292.1027 Da. If at least one GlcNAc oxonium ion was detected with 15 ppm mass accuracy, the corresponding precursor ion was used to collect an EThcD spectrum in the orbitrap at R of 30,000. For charges of 2 and 3, electron transfer dissociation (ETD) target was 5.0 × 10^5^; for charges of 4 to 8, ETD target was 2.0 × 10^5^. Supplemental collision energy at 15% was also included. Reaction time of ETD was variable according to the precursor charge state. For a charge of 2, ETD reaction time was 125 msec; for a charge of 3, ETD reaction time was 100 msec; for a charge of 4, ETD reaction time was 75 msec; and for charges ≥5, ETD reaction time was 50 msec.

### MS data analysis to determine O-GlcNAc modification sites

RAW data files were analyzed with the MetaMorpheus software version 1.0.1 (https://github.com/smith-chem-wisc/MetaMorpheus) developed by the Smith laboratory (126). For hT40 proteins, the following FASTA files were downloaded from UniProt (November 2021) and used for analysis: *Escherichia coli* (strain K12) (UP000000625), Asp-N (Q9R4J4), Lys-C (Q02SZ7), and full-length tau (2N4R isoform, P10636-8). The same FASTA files were used to analyze the hT39 proteins except full-length tau (2N4R isoform, P10636-8) was replaced with 2N3R tau isoform (P10636-5). A mass shift of +203.079 Da (C_8_H_13_NO_5_) was used to search for O-GlcNAc modifications (71) on S and T. In addition, the following *m/z* corresponding to diagnostic ions were investigated: +126.055 Da (C_6_H_7_NO_2_), +138.055 Da (C_7_H_7_NO_2_), +144.066 Da (C_6_H_9_NO_3_), +168.066 Da (C_8_H_9_NO_3_), +186.076 Da (C_8_H_11_NO_4_), and +204.087 Da (C_8_H_13_NO_5_) (71).

The analysis sequence included mass calibration, global post-translational modification discovery (G-PTM-D) (127), and a classic search. Mass calibration was conducted using the following criteria: protease = Asp-N/Lys-C; maximum missed cleavages = 2; minimum peptide length = 7; maximum peptide length = unspecified; initiator methionine behavior = variable; variable modifications = oxidation on M; max mods per peptide = 2; max modification isoforms = 1024; precursor mass tolerance = ±15.0000 PPM; product mass tolerance = ±25.0000 PPM. The criteria utilized for G-PTM-D were protease = Asp-N/Lys-C; maximum missed cleavages = 2; minimum peptide length = 7; maximum peptide length = unspecified; initiator methionine behavior = variable; max modification isoforms = 1024; variable modifications = oxidation on M; G-PTM-D modifications count = 3; precursor mass tolerance(s) = ±5.0000 PPM around 0, 203.079372521 Da; and product mass tolerance = ±20.0000 PPM. Finally, a classic search was conducted using the following criteria: protease = Asp-N/Lys-C; search for truncated proteins and proteolysis products = false; maximum missed cleavages = 2; minimum peptide length = 7; maximum peptide length = unspecified; initiator methionine behavior = variable; variable modifications = oxidation on M; precursor mass tolerance = ±5.0000 PPM; product mass tolerance = ±20.0000 PPM; report peptide spectral match (PSM) ambiguity = true. Peptides were quantified through the FlashLFQ method for label-free quantification bundled into MetaMorpheus (128). At least two peptides were required to identify the protein. Sites of O-GlcNAc modification on tau detected at a false discovery rate (calculated using the target-decoy approach) of 1% are reported (Table S1). Table S2 contains all quantified tau peptides in unmodified *versus* GlcNAc-modified tau samples. Table S3 shows the quantified peaks of tau with their corresponding peptide masses, theoretical and observed *m/z*, retention time, and PSMs. MetaDraw version 1.0.5 was utilized to review the PSMs of modified and unmodified tau peptides (samples of these peptides are included in Figs. S1 and S2). Processed proteomics data on tau peptides are available in this article (Tables S1–S3 and Supporting Information) and full proteomics datasets and .RAW files from MS are available in a public repository (https://doi.org/10.5061/dryad.m0cfxppdc).

### Tubulin polymerization assay

Tubulin polymerization in the presence of recombinant tau proteins was assessed using the Tubulin Polymerization Assay Kit (Cytoskeleton, #BK011P). Kit reagents were reconstituted and stored as indicated in the manual. The 96-well plate provided with the kit was incubated at 37 °C for 10 min (Synergy H1 Hybrid Multi-Mode Reader and Gen5 software v3.11, BioTek; https://www.agilent.com/en/product/microplate-instrumentation/microplate-instrumentation-control-analysis-software/imager-reader-control-analysis-software). Recombinant tau proteins were prepared as 10 μM stocks in general tubulin buffer (80 mM Pipes pH 6.9, 2 mM MgCl_2_, and 0.5 mM EGTA) and left at RT. Then, a tubulin master mix was prepared and kept on ice using the following recipe (kit manual): 355 μl of buffer 1, 4.4 μl of 100 mM GTP, and 85 μl of tubulin 10 mg/ml. Once the 10-min incubation of the 96-well plate was over, recombinant tau (10 μM stocks) were loaded (5 μl) onto the plate and warmed to 37 °C for exactly 1 min. Then, the tubulin master mix was loaded (50 μl) on each tau protein sample yielding a final tau concentration of 1 μM. Fluorescence signal was measured for 1 h to monitor tubulin polymerization using the kinetic mode at excitation and emission wavelengths of 360 nm and 450 nm, respectively. Each tau protein sample was loaded in duplicate (technical replicates) with a sample size of 4 (independent biological replicates). Background levels of blank (general tubulin buffer only) were subtracted from the fluorescence readings before further analyzing the data. Nonlinear regression using “specific binding with Hill slope” was used to fit the tubulin polymerization data in GraphPad Prism v10.2.1 (https://www.graphpad.com/features). The polymerization rate or time to half maximal polymerization (K_d_) and the steady-state equilibrium (V_max_) were calculated.

### Microtubule-binding assay

Assays were performed using Microtubule Binding Protein Spin-Down Assay Biochem Kit (Cytoskeleton, #BK029). Kit reagents were reconstituted as indicated in the manual. Tubulin aliquots (20 μl) were thawed, supplemented with cushion buffer (2 μl; 80 mM PIPES pH 7.0, 1 mM MgCl_2_, 1 mM EGTA, and 60% sucrose), and incubated at 35 °C for exactly 40 min. Then, general tubulin buffer (200 μl; 80 mM PIPES pH 7.0, 1 mM MgCl_2_, and 1 mM EGTA) supplemented with paclitaxel at 20 μM was added to the polymerized tubulin. Microtubule-binding reactions were set up at RT including tubulin (10 μl), recombinant tau proteins (1 μM), and general tubulin buffer to a final volume of 50 μl. After 30 min of incubation at RT, binding reactions were loaded on 100 μl of sucrose cushion buffer in 0.2 ml polycarbonate tubes (Thermo Fisher Scientific, #45233) followed by centrifugation at 100,000 RCF for 40 min. For all centrifugation steps, S100-AT3 Fixed Angle Rotor (Thermo Fisher Scientific, #45585) and Sorvall MTX 150 Micro-Ultracentrifuge (Thermo Fisher Scientific, #46960) were used. Then, 30 μl of the supernatant were carefully removed (Sup) to avoid disturbing the microtubule pellet. Laemmli buffer was added to the supernatant immediately (6 μl of 6X Laemmli buffer). The rest of the supernatant was discarded, followed by washing the microtubule pellet with general tubulin buffer (100 μl) supplemented with 20 μM paclitaxel. The microtubule pellet was subjected to centrifugation at 100,000 RCF for an additional 20 min. The previous washing step was repeated one more time, followed by careful removal of the supernatant. The final pellet was resuspended in 60 μl of 1X Laemmli buffer. The assay was conducted four independent times.

For each biological replicate, Sup and Pellet (15 μl each) were subjected to SDS-PAGE. Unmodified and Glc tau samples were loaded on two separate 4 to 20% Novex tris-glycine gels (Invitrogen, #WXP42020) and run at 225 V for 40 to 45 min. After SDS-PAGE, the two gels were cut between the 250 and 37 kDa marker bands. Two gels were added to the same transfer sandwich and transferred onto one nitrocellulose membrane. This step allows for the quantification of tau bands from two experimental replicates on the same blot to minimize the variability in blotting procedure across samples. The rest of the blotting procedure was performed as described earlier. Primary antibodies used were the Tau5 antibody at 1:100,000 (same as above) and the tubulin 5H1 antibody at 1:10,000 (Nicholas M. Kanaan at Michigan State University, RRID: AB_2832941) (129). Secondary antibodies used were goat anti-mouse IgG1 680 (LI-COR Biosciences, #926-68050, RRID: AB_2783642) and goat anti-mouse IgM 800 (LI-COR Biosciences, #926-32280, RRID: AB_2814919) at 1:20,000 each. LI-COR Odyssey classic imager and Image Studio Lite Ver 5.2 (https://www.licorbio.com/image-studio) were used to visualize fluorescent signals of the antibody probes. Tau bands were quantified in all Sup and Pellet fractions followed by calculating the percentage of tau in Pellet relative to total tau using the following equation.

$$\%\;Tau\;in\;Pellet=\frac{Tau\;in\;Pellet}{\left(Tau\;in\;Pellet+Tau\;in\;\mathrm{Sup}\right)}\;x\;100$$

### *In vitro* recombinant tau aggregation reaction

Aggregation of tau proteins was induced by arachidonic acid (ARA; Cayman Chemical, #90010) in 200 μl reactions as described previously (95, 130). Briefly, 2 μM of tau (M. Wt. = 43,426 for hT39; M. Wt. = 46,673 for hT40) was induced to aggregate *in vitro* by incubation in tau aggregation buffer (10 mM sodium Hepes, 0.1 mM EDTA, 200 mM NaCl, 5 mM DTT, pH 7.6) with (aggregated) or without (unaggregated; ethanol vehicle was used) 75 μM ARA. ARA stocks were prepared in 100% ethanol at 2 mM immediately prior to use. Finally, ARA was added as the final component in the reaction sample and then the samples were gently mixed by minimally shaking the tube/cuvette. Aggregation reactions were incubated at RT for 6 h.

### Right-angle LLS

As tau polymerizes into aggregates, the intensity of scattered light increases as a function of time and is used to measure tau aggregation kinetics (95, 131). LLS was measured using class IIb laser with wavelength of 475 nm and maximum power of 20 mW (B & W INC., model #BWI-475-20-E) and digital camera (Thor Labs, model #DCC1240M). Images were acquired using uc480 Viewer version 4.2 with the pixel clock set at 11 MHz. Images were acquired for hT40 samples at a frame rate of 2 fps and exposure of 150 ms, while a frame rate of 1 fps and exposure time to 300 ms was used for hT39 samples. Polymerization reactions of tau were transferred into glass cuvettes with path length of 5 mm (Starna Cells, #3-G-5), and LLS was measured at time zero, prior to addition of ARA to obtain baseline measurements. After addition of ARA and gently mixing the samples, images were serially acquired at 1, 2, 3, 4, 5, 10, 15, 20, 25, 30, 35, 40, 45, 50, 55, 60, 75, 90, 105, 120, 150, 180, 240, 300, and 360 min. Each experiment was conducted four independent times. Image analysis was performed with Adobe Photoshop CS6 (Adobe Systems INC.) using the marquee tool. The region of interest used for densitometry measurements was set to 150 pixels × 15 pixels and adjusted to the center of the glass cuvette within the band of scattered light. Pixel intensity was recorded using the histogram feature. Scattered light intensity (Is) measurements during the 6 h were fitted using nonlinear Finke–Watzky function (132) according to the following equation:

$${\left[B\right]}_{t}={\left[A\right]}_{0}\left(1-\frac{k_{1}+k_{2}{\left[A\right]}_{0}}{k_{2}{\left[A\right]}_{0}+k_{1}\cdot e^{\left(k_{1}+k_{2}{\left[A\right]}_{0}\right)t}}\right)$$

To compare aggregation kinetics of different proteins, the following parameters were calculated: k_1_ represents the rate constant of nucleation; k_2_ represents the rate constant of elongation; and [A]_0_ represents maximum scattering.

### ThS fluorescence

At the end of the tau aggregation reaction with ARA (described above), β-sheet–containing aggregates were quantified using a ThS assay as described (95). Immediately before starting the assay, a 0.0175% ThS solution was prepared in water, filtered through a 0.22 μm membrane, and protected from light. Then, 150 μl of each tau sample was mixed with 6 μl of ThS solution and incubated for 5 min at RT. The samples (150 μl/well) were then loaded onto a black 96-well plate (Costar, #3915) and immediately read using the Promega Glomax multidetection system at 490 nm excitation and 510 to 570 nm emission wavelengths. Control buffers for unaggregated and aggregated reactions were loaded and read to obtain background measurements, and then their absorbance values were subtracted from the other values before analysis.

### Quantitative measurements of tau aggregation using TEM

TEM was used to visualize and measure aggregate density and size (95). To this end, uranyl acetate (UA; Electron Microscopy Sciences, #22400) solution was freshly prepared by dissolving 20 mg of UA in 1 ml of deionized water (2% UA solution) at RT. After UA completely dissolves into deionized water, the solution was sterile filtered using 0.22 μm membrane (Thermo Fisher Scientific, #13-1001-06). To prepare the grids, each unaggregated and aggregated tau sample (10 μl each) was fixed with 2% glutaraldehyde (Electron Microscopy Sciences, #16100) at RT for 10 min. Then, each sample (5 μl) was absorbed onto formvar-coated copper grids (Electron Microscopy Sciences, #FCF300-CU) for 1 min, followed by one rapid rinse in diagnostic ion water and another rapid rinse in UA solution. Finally, the grids were incubated with UA solution for 1 min at RT, then the solution was wicked away, and grids were left to dry for at least 1.5 h before imaging. Grids were prepared from four independent replicates of unaggregated and aggregated tau grids and imaged using JEOL JEM-1400 Plus electron microscope at 80 kV. Electron micrographs were captured and saved through AMT XR81 digital camera and AMT software version 602.6 (https://amtimaging.com/home).

For each grid, three images were captured at 5000X magnification for quantitative TEM. Electron micrographs were then processed using ImageJ v1.54 (https://imagej.net/ij/download.html) using a method like that described by Tiernan *et al.* (95). First, the image scale was adjusted according to the scale bar attached to the TEM images. Then, images were smoothened three times to allow for the automatic thresholding to capture the visible tau aggregates. Finally, the percentage area of aggregated mass (% Area) was collected by the “Analyze Particles” command. For the “Analyze Particles” command, size was set at 0 to infinity and circularity was set at 0 to 1.

Output of “Analyze Particles” command was further processed using GraphPad Prism v10.2.1 to obtain frequency distribution using 700 nm^2^-wide and 50 nm^2^-wide bins for hT40 and hT39, respectively. The sum of particles in three images/replicate was calculated and counted as 1 independent replicate, with a total of four independent replicates. The following populations of hT40 were plotted according to criteria like those reported by Tiernan *et al.* (95): <700 nm^2^ for globular aggregates only; 700 to 2100 nm^2^ for globular aggregates >700 nm^2^ along with short filaments; 2100 to 5000 nm^2^ for short filaments only; and >5000 nm^2^ for long filaments only. Aggregates of hT39 were globular in nature and split into sizes smaller and larger than 1200 nm^2^.

### Western blot for unaggregated and aggregated reactions

Unaggregated and aggregated tau samples were prepared for SDS-PAGE by diluting to 0.5 μM in 6X Laemmli sample buffer. Samples were boiled at 95 °C for 5 min in a heat block, followed by vortex and quick spin down. Samples (500 ng/lane) were loaded on 20-well 4 to 20% Novex tris-glycine gel (Invitrogen, #WXP42020). Proteins were transferred to nitrocellulose membrane using the Bio-Rad wet transfer system. The blot was blocked with 2% NFDM for 1 h at RT, followed by incubation in Tau5 antibody (as above) at a dilution of 1:100,000 in 2% NFDM overnight at 4 °C. The following day, the membrane was washed three times with TBST, 5 min each. The goat anti-mouse IgG1 680 (as above) secondary antibody was used at a dilution of 1:20,000 in 2% NFDM, and membranes were incubated in secondary at RT for 1 h. Membranes were washed with TBST three times, 5 min each, before imaging the blot using LI-COR Odyssey classic imager and Image Studio Lite Ver 5.2. Tau bands corresponding to monomeric tau (monomer band) and higher molecular weight multimers (HMW band) were quantified in the unaggregated and aggregated tau samples.

### Sandwich ELISA

To detect pathological tau conformations using conformation-dependent antibodies (*i*.*e*., TOC1, TOMA1, TNT2, and Alz50), tau samples must be kept under native conditions (38, 48, 58, 64, 122). Therefore, sandwich ELISA assays were employed to measure pathological tau conformations. All steps were performed at RT. The following capture antibodies were used in sandwich ELISA assays: TOC1 (Nicholas M. Kanaan at Michigan State University, RRID: AB_2832939) to measure TOC1-positive oligomeric tau, TOMA1 (Millipore, #MABN819) to measure TOMA1-positive oligomeric tau, TNT2 (Nicholas M. Kanaan at Michigan State University, RRID: AB_2736931) to measure PAD exposed tau, Alz50 (P. Davies Albert Einstein College of Medicine, RRID: AB_2313937) to measure misfolded tau, and Tau13 (Nicholas M. Kanaan at Michigan State University, RRID: AB_2721193) to measure total tau (36, 38, 42, 48, 52, 58, 59, 64, 66). Capture antibodies were diluted to 2 ng/μl in borate saline buffer (100 mM borate acid, 25 mM sodium borate, 75 mM NaCl, and 0.25 mM thimerosal). Then, sandwich ELISA plates (96-well plates, Corning, #3590) were coated with the capture antibodies (50 μl/well) for 1 h. Wells were washed two times with ELISA wash buffer (200 μl/well; 100 mM borate acid, 25 mM sodium borate, 75 mM NaCl, 0.25 mM thimerosal, 0.4% (w/v) bovine serum albumin, and 0.05% (v/v) Tween-20), followed by blocking with 5% NFDM in ELISA wash buffer (200 μl/well) for 1 h. Two washes with ELISA wash buffer were performed, followed by the addition of unaggregated and aggregated tau samples for 1.5 h. Tau samples were prepared from unaggregated and aggregated hT40 reactions in TBS buffer at the following concentrations (50 μl/well): 2.5 nM for Tau13; 5 nM for TNT2; 20 nM for TOC1 and Alz50; and 150 nM for TOMA1. Tau samples were prepared from unaggregated and aggregated hT39 reactions in TBS buffer at the following concentrations (50 μl/well): 2.5 nM for Tau13; 20 nM for Alz50; 50 nM for TNT2; and 150 nM for TOC1 and TOMA1. Then, wells were washed four times with ELISA wash buffer (200 μl/well). The detection antibody R1 (Nicholas M. Kanaan at Michigan State University, RRID: AB_2832929) (133) was diluted at 1:10,000 in 5% NFDM and added to the wells (50 μl/well) for 1.5 h. Then, sandwich ELISA wells were washed four times with ELISA wash buffer. The secondary antibody used was peroxidase-conjugated goat anti-rabbit IgG antibody (H + L; Vector Laboratories, #PI-1000-1, RRID: AB_2916034) at 1:5000 in 5% NFDM for 1 h (50 μl/well). After four final washes with ELISA wash buffer, the peroxidase reaction was developed using 3,3′,5,5′-tetramethylbenzidine (50 μl/well; Sigma, #T0440). The peroxidase reaction was stopped with 4% sulphuric acid, followed by reading the absorbance at 450 nm using SpectraMax Plus 384 microplate reader (Molecular Devices). Each assay was run using four independent experimental samples.

The absorbance values were further processed using GraphPad Prism v10.2.1 according to the following equation to calculate the percentage light absorbed (% Light Abs, %A).

$$\%A=100-\left(100*10^{-A}\right)$$

### Statistics

Statistical analysis was performed using GraphPad Prism v10.2.1. Unpaired, two-tailed *t* test was employed to analyze the following results: V_max_ and K_d_ of tubulin polymerization assay; Max and k_1_ of LLS for hT40; Max of LLS for hT39; % area of quantitative TEM. Mann–Whitney *U* was used for violations of normality and/or equal variances: k_2_ of LLS data for hT40; k_1_ and k_2_ of LLS data for hT39. Two-way ANOVA followed by the *post hoc* Holm–Sidak with all possible comparisons were used to analyze the following results: % Tau in pellet of microtubule-binding assay; ThS fluorescence; monomer and high molecular weight bands for stable multimers; and % light absorbed of sandwich ELISA assays. Differences in outcomes were deemed statistically significant at *p* ≤ 0.05.

## Data availability

All the data are provided in this article, the supplemental information, and/or on Dryad (https://doi.org/10.5061/dryad.m0cfxppdc). Any additional information is available upon reasonable request to Dr Nicholas Kanaan (corresponding author) at nkanaan@msu.edu.

## Supporting information

This article contains supporting information.

## Conflicts of interest

The authors declare that they have no conflicts of interest with the contents of this article.

## References

[1] Gotz J., Halliday G., Nisbet R.M. (2019). Molecular pathogenesis of the tauopathies. Annu. Rev. Pathol..

[2] McKee A.C., Stein T.D., Kiernan P.T., Alvarez V.E. (2015). The neuropathology of chronic traumatic encephalopathy. Brain Pathol..

[3] McKee A.C., Stern R.A., Nowinski C.J., Stein T.D., Alvarez V.E., Daneshvar D.H. (2013). The spectrum of disease in chronic traumatic encephalopathy. Brain.

[4] McKee A.C., Abdolmohammadi B., Stein T.D. (2018). The neuropathology of chronic traumatic encephalopathy. Handb Clin. Neurol..

[5] Wang Y., Mandelkow E. (2016). Tau in physiology and pathology. Nat. Rev. Neurosci..

[6] Alhadidy M.M., Kanaan N.M. (2024). Biochemical approaches to assess the impact of post-translational modifications on pathogenic tau conformations using recombinant protein. Biochem. Soc. Trans..

[7] Arendt T., Stieler J.T., Holzer M. (2016). Tau and tauopathies. Brain Res. Bull.

[8] Ye H., Han Y., Li P., Su Z., Huang Y. (2022). The role of post-translational modifications on the structure and function of tau protein. J. Mol. Neurosci..

[9] Park S., Lee J.H., Jeon J.H., Lee M.J. (2018). Degradation or aggregation: the ramifications of post-translational modifications on tau. BMB Rep..

[10] Alquezar C., Arya S., Kao A.W. (2020). Tau post-translational modifications: dynamic transformers of tau function, degradation, and aggregation. Front. Neurol..

[11] Ferrer I., Lopez-Gonzalez I., Carmona M., Arregui L., Dalfo E., Torrejon-Escribano B. (2014). Glial and neuronal tau pathology in tauopathies: characterization of disease-specific phenotypes and tau pathology progression. J. Neuropathol. Exp. Neurol..

[12] Cook C., Stankowski J.N., Carlomagno Y., Stetler C., Petrucelli L. (2014). Acetylation: a new key to unlock tau's role in neurodegeneration. Alzheimers Res. Ther..

[13] Yang X., Qian K. (2017). Protein O-GlcNAcylation: emerging mechanisms and functions. Nat. Rev. Mol. Cell. Biol..

[14] Arnold C.S., Johnson G.V., Cole R.N., Dong D.L., Lee M., Hart G.W. (1996). The microtubule-associated protein tau is extensively modified with O-linked N-acetylglucosamine. J. Biol. Chem..

[15] Raut S., Bhalerao A., Powers M., Gonzalez M., Mancuso S., Cucullo L. (2023). Hypometabolism, Alzheimer's disease, and possible therapeutic targets: an overview. Cells.

[16] Ibanez V., Pietrini P., Alexander G.E., Furey M.L., Teichberg D., Rajapakse J.C. (1998). Regional glucose metabolic abnormalities are not the result of atrophy in Alzheimer's disease. Neurology.

[17] De Santi S., de Leon M.J., Rusinek H., Convit A., Tarshish C.Y., Roche A. (2001). Hippocampal formation glucose metabolism and volume losses in MCI and AD. Neurobiol. Aging.

[18] de Leon M.J., Convit A., Wolf O.T., Tarshish C.Y., DeSanti S., Rusinek H. (2001). Prediction of cognitive decline in normal elderly subjects with 2-[(18)F]fluoro-2-deoxy-D-glucose/poitron-emission tomography (FDG/PET). Proc. Natl. Acad. Sci. U. S. A..

[19] Costantini L.C., Barr L.J., Vogel J.L., Henderson S.T. (2008). Hypometabolism as a therapeutic target in Alzheimer's disease. BMC Neurosci..

[20] Liu F., Shi J., Tanimukai H., Gu J., Gu J., Grundke-Iqbal I. (2009). Reduced O-GlcNAcylation links lower brain glucose metabolism and tau pathology in Alzheimer's disease. Brain.

[21] Liu F., Iqbal K., Grundke-Iqbal I., Hart G.W., Gong C.X. (2004). O-GlcNAcylation regulates phosphorylation of tau: a mechanism involved in Alzheimer's disease. Proc. Natl. Acad. Sci. U. S. A..

[22] Gong C.X., Liu F., Iqbal K. (2016). O-GlcNAcylation: a regulator of tau pathology and neurodegeneration. Alzheimers Dement.

[23] Yuzwa S.A., Shan X., Macauley M.S., Clark T., Skorobogatko Y., Vosseller K. (2012). Increasing O-GlcNAc slows neurodegeneration and stabilizes tau against aggregation. Nat. Chem. Biol..

[24] Yuzwa S.A., Cheung A.H., Okon M., McIntosh L.P., Vocadlo D.J. (2014). O-GlcNAc modification of tau directly inhibits its aggregation without perturbing the conformational properties of tau monomers. J. Mol. Biol..

[25] Smet-Nocca C., Broncel M., Wieruszeski J.M., Tokarski C., Hanoulle X., Leroy A. (2011). Identification of O-GlcNAc sites within peptides of the Tau protein and their impact on phosphorylation. Mol. Biosyst..

[26] Gatta E., Lefebvre T., Gaetani S., dos Santos M., Marrocco J., Mir A.M. (2016). Evidence for an imbalance between tau O-GlcNAcylation and phosphorylation in the hippocampus of a mouse model of Alzheimer's disease. Pharmacol. Res..

[27] Yuzwa S.A., Macauley M.S., Heinonen J.E., Shan X., Dennis R.J., He Y. (2008). A potent mechanism-inspired O-GlcNAcase inhibitor that blocks phosphorylation of tau in vivo. Nat. Chem. Biol..

[28] Wang X., Li W., Marcus J., Pearson M., Song L., Smith K. (2020). MK-8719, a novel and selective O-GlcNAcase inhibitor that reduces the formation of pathological tau and ameliorates neurodegeneration in a mouse model of tauopathy. J. Pharmacol. Exp. Ther..

[29] Hastings N.B., Wang X., Song L., Butts B.D., Grotz D., Hargreaves R. (2017). Inhibition of O-GlcNAcase leads to elevation of O-GlcNAc tau and reduction of tauopathy and cerebrospinal fluid tau in rTg4510 mice. Mol. Neurodegener..

[30] Graham D.L., Gray A.J., Joyce J.A., Yu D., O'Moore J., Carlson G.A. (2014). Increased O-GlcNAcylation reduces pathological tau without affecting its normal phosphorylation in a mouse model of tauopathy. Neuropharmacology.

[31] Fitzpatrick A.W.P., Falcon B., He S., Murzin A.G., Murshudov G., Garringer H.J. (2017). Cryo-EM structures of tau filaments from Alzheimer's disease. Nature.

[32] Zhang W., Tarutani A., Newell K.L., Murzin A.G., Matsubara T., Falcon B. (2020). Novel tau filament fold in corticobasal degeneration. Nature.

[33] Falcon B., Zhang W., Murzin A.G., Murshudov G., Garringer H.J., Vidal R. (2018). Structures of filaments from Pick's disease reveal a novel tau protein fold. Nature.

[34] Shi Y., Zhang W., Yang Y., Murzin A.G., Falcon B., Kotecha A. (2021). Structure-based classification of tauopathies. Nature.

[35] Zhang W., Falcon B., Murzin A.G., Fan J., Crowther R.A., Goedert M. (2019). Heparin-induced tau filaments are polymorphic and differ from those in Alzheimer's and Pick's diseases. Elife.

[36] Patterson K.R., Remmers C., Fu Y., Brooker S., Kanaan N.M., Vana L. (2011). Characterization of prefibrillar Tau oligomers in vitro and in Alzheimer disease. J. Biol. Chem..

[37] Lasagna-Reeves C.A., Castillo-Carranza D.L., Sengupta U., Sarmiento J., Troncoso J., Jackson G.R. (2012). Identification of oligomers at early stages of tau aggregation in Alzheimer's disease. FASEB J.

[38] Combs B., Hamel C., Kanaan N.M. (2016). Pathological conformations involving the amino terminus of tau occur early in Alzheimer's disease and are differentially detected by monoclonal antibodies. Neurobiol. Dis..

[39] Weaver C.L., Espinoza M., Kress Y., Davies P. (2000). Conformational change as one of the earliest alterations of tau in Alzheimer's disease. Neurobiol. Aging.

[40] Niewiadomska G., Niewiadomski W., Steczkowska M., Gasiorowska A. (2021). Tau oligomers neurotoxicity. Life (Basel).

[41] Mroczko B., Groblewska M., Litman-Zawadzka A. (2019). The role of protein misfolding and tau oligomers (TauOs) in Alzheimer's disease (AD). Int. J. Mol. Sci..

[42] Garcia-Sierra F., Ghoshal N., Quinn B., Berry R.W., Binder L.I. (2003). Conformational changes and truncation of tau protein during tangle evolution in Alzheimer's disease. J. Alzheimers Dis..

[43] Cox K., Combs B., Abdelmesih B., Morfini G., Brady S.T., Kanaan N.M. (2016). Analysis of isoform-specific tau aggregates suggests a common toxic mechanism involving similar pathological conformations and axonal transport inhibition. Neurobiol. Aging.

[44] Castillo-Carranza D.L., Gerson J.E., Sengupta U., Guerrero-Munoz M.J., Lasagna-Reeves C.A., Kayed R. (2014). Specific targeting of tau oligomers in Htau mice prevents cognitive impairment and tau toxicity following injection with brain-derived tau oligomeric seeds. J. Alzheimers Dis..

[45] Montalbano M., Majmundar L., Sengupta U., Fung L., Kayed R. (2023). Pathological tau signatures and nuclear alterations in neurons, astrocytes and microglia in Alzheimer's disease, progressive supranuclear palsy, and dementia with Lewy bodies. Brain Pathol..

[46] Kanaan N.M., Cox K., Alvarez V.E., Stein T.D., Poncil S., McKee A.C. (2016). Characterization of early pathological tau conformations and phosphorylation in chronic traumatic encephalopathy. J. Neuropathol. Exp. Neurol..

[47] Santacruz K., Lewis J., Spires T., Paulson J., Kotilinek L., Ingelsson M. (2005). Tau suppression in a neurodegenerative mouse model improves memory function. Science.

[48] Castillo-Carranza D.L., Sengupta U., Guerrero-Munoz M.J., Lasagna-Reeves C.A., Gerson J.E., Singh G. (2014). Passive immunization with Tau oligomer monoclonal antibody reverses tauopathy phenotypes without affecting hyperphosphorylated neurofibrillary tangles. J. Neurosci..

[49] Van der Jeugd A., Hochgrafe K., Ahmed T., Decker J.M., Sydow A., Hofmann A. (2012). Cognitive defects are reversible in inducible mice expressing pro-aggregant full-length human Tau. Acta. Neuropathol..

[50] Sydow A., Van der Jeugd A., Zheng F., Ahmed T., Balschun D., Petrova O. (2011). Tau-induced defects in synaptic plasticity, learning, and memory are reversible in transgenic mice after switching off the toxic Tau mutant. J. Neurosci..

[51] Kanaan N.M., Morfini G.A., LaPointe N.E., Pigino G.F., Patterson K.R., Song Y. (2011). Pathogenic forms of tau inhibit kinesin-dependent axonal transport through a mechanism involving activation of axonal phosphotransferases. J. Neurosci..

[52] Combs B., Kanaan N.M. (2017). Exposure of the amino terminus of tau is a pathological event in multiple tauopathies. Am. J. Pathol..

[53] Hintermayer M.A., Volkening K., Moszczynski A.J., Donison N., Strong M.J. (2020). Tau protein phosphorylation at Thr(175) initiates fibril formation via accessibility of the N-terminal phosphatase-activating domain. J. Neurochem..

[54] Combs B., Christensen K.R., Richards C., Kneynsberg A., Mueller R.L., Morris S.L. (2021). Frontotemporal lobar dementia mutant tau impairs axonal transport through a protein phosphatase 1gamma-dependent mechanism. J. Neurosci..

[55] Christensen K.R., Combs B., Richards C., Grabinski T., Alhadidy M.M., Kanaan N.M. (2023). Phosphomimetics at Ser199/Ser202/Thr205 in tau impairs axonal transport in rat hippocampal neurons. Mol. Neurobiol..

[56] Mueller R.L., Combs B., Alhadidy M.M., Brady S.T., Morfini G.A., Kanaan N.M. (2021). Tau: a signaling hub protein. Front. Mol. Neurosci..

[57] Morris S.L., Brady S.T. (2022). Tau phosphorylation and PAD exposure in regulation of axonal growth. Front. Cell Dev. Biol..

[58] Jicha G.A., Bowser R., Kazam I.G., Davies P. (1997). Alz-50 and MC-1, a new monoclonal antibody raised to paired helical filaments, recognize conformational epitopes on recombinant tau. J. Neurosci. Res..

[59] Carmel G., Mager E.M., Binder L.I., Kuret J. (1996). The structural basis of monoclonal antibody Alz50's selectivity for Alzheimer's disease pathology. J. Biol. Chem..

[60] Ksiezak-Reding H., Davies P., Yen S.H. (1988). Alz 50, a monoclonal antibody to Alzheimer's disease antigen, cross-reacts with tau proteins from bovine and normal human brain. J. Biol. Chem..

[61] Kanaan N.M., Hamel C., Grabinski T., Combs B. (2020). Liquid-liquid phase separation induces pathogenic tau conformations in vitro. Nat. Commun..

[62] Jeganathan S., Hascher A., Chinnathambi S., Biernat J., Mandelkow E.M., Mandelkow E. (2008). Proline-directed pseudo-phosphorylation at AT8 and PHF1 epitopes induces a compaction of the paperclip folding of Tau and generates a pathological (MC-1) conformation. J. Biol. Chem..

[63] Tiernan C.T., Mufson E.J., Kanaan N.M., Counts S.E. (2018). Tau oligomer pathology in nucleus basalis neurons during the progression of Alzheimer disease. J. Neuropathol. Exp. Neurol..

[64] Ward S.M., Himmelstein D.S., Lancia J.K., Fu Y., Patterson K.R., Binder L.I. (2013). TOC1: characterization of a selective oligomeric tau antibody. J. Alzheimers Dis..

[65] LaPointe N.E., Morfini G., Pigino G., Gaisina I.N., Kozikowski A.P., Binder L.I. (2009). The amino terminus of tau inhibits kinesin-dependent axonal transport: implications for filament toxicity. J. Neurosci. Res..

[66] Hyman B.T., Van Hoesen G.W., Wolozin B.L., Davies P., Kromer L.J., Damasio A.R. (1988). Alz-50 antibody recognizes Alzheimer-related neuronal changes. Ann. Neurol..

[67] Yuzwa S.A., Yadav A.K., Skorobogatko Y., Clark T., Vosseller K., Vocadlo D.J. (2011). Mapping O-GlcNAc modification sites on tau and generation of a site-specific O-GlcNAc tau antibody. Amino Acids.

[68] Morris M., Knudsen G.M., Maeda S., Trinidad J.C., Ioanoviciu A., Burlingame A.L. (2015). Tau post-translational modifications in wild-type and human amyloid precursor protein transgenic mice. Nat. Neurosci..

[69] Cantrelle F.X., Loyens A., Trivelli X., Reimann O., Despres C., Gandhi N.S. (2021). Phosphorylation and O-GlcNAcylation of the PHF-1 epitope of tau protein induce local conformational changes of the C-terminus and modulate tau self-assembly into fibrillar aggregates. Front. Mol. Neurosci..

[70] El Mammeri N., Dregni A.J., Duan P., Wang H.K., Hong M. (2022). Microtubule-binding core of the tau protein. Sci. Adv..

[71] Ma J., Hart G.W. (2017). Analysis of protein O-GlcNAcylation by mass spectrometry. Curr Protoc Protein Sci..

[72] Wesseling H., Mair W., Kumar M., Schlaffner C.N., Tang S., Beerepoot P. (2020). Tau PTM profiles identify patient heterogeneity and stages of Alzheimer's disease. Cell.

[73] Xia Y., Prokop S., Giasson B.I. (2021). "Don't Phos over Tau": recent developments in clinical biomarkers and therapies targeting tau phosphorylation in Alzheimer's disease and other tauopathies. Mol. Neurodegener.

[74] Suarez-Calvet M., Karikari T.K., Ashton N.J., Lantero Rodriguez J., Mila-Aloma M., Gispert J.D. (2020). Novel tau biomarkers phosphorylated at T181, T217 or T231 rise in the initial stages of the preclinical Alzheimer's continuum when only subtle changes in Abeta pathology are detected. EMBO Mol. Med..

[75] Moszczynski A.J., Yang W., Hammond R., Ang L.C., Strong M.J. (2017). Threonine(175), a novel pathological phosphorylation site on tau protein linked to multiple tauopathies. Acta. Neuropathol. Commun..

[76] Jack C.R., Bennett D.A., Blennow K., Carrillo M.C., Dunn B., Haeberlein S.B. (2018). NIA-AA Research Framework: toward a biological definition of Alzheimer's disease. Alzheimers Dement.

[77] Barthelemy N.R., Mallipeddi N., Moiseyev P., Sato C., Bateman R.J. (2019). Tau phosphorylation rates measured by mass spectrometry differ in the intracellular brain vs. Extracellular cerebrospinal fluid compartments and are differentially affected by Alzheimer's disease. Front. Aging Neurosci..

[78] Otvos L., Feiner L., Lang E., Szendrei G.I., Goedert M., Lee V.M. (1994). Monoclonal antibody PHF-1 recognizes tau protein phosphorylated at serine residues 396 and 404. J. Neurosci. Res..

[79] Guillozet-Bongaarts A.L., Cahill M.E., Cryns V.L., Reynolds M.R., Berry R.W., Binder L.I. (2006). Pseudophosphorylation of tau at serine 422 inhibits caspase cleavage: in vitro evidence and implications for tangle formation in vivo. J. Neurochem..

[80] Vana L., Kanaan N.M., Ugwu I.C., Wuu J., Mufson E.J., Binder L.I. (2011). Progression of tau pathology in cholinergic Basal forebrain neurons in mild cognitive impairment and Alzheimer's disease. Am. J. Pathol..

[81] Mondragon-Rodriguez S., Perry G., Luna-Munoz J., Acevedo-Aquino M.C., Williams S. (2014). Phosphorylation of tau protein at sites Ser(396-404) is one of the earliest events in Alzheimer's disease and down syndrome. Neuropathol. Appl. Neurobiol..

[82] Kimura T., Ono T., Takamatsu J., Yamamoto H., Ikegami K., Kondo A. (1996). Sequential changes of tau-site-specific phosphorylation during development of paired helical filaments. Dementia.

[83] Guillozet-Bongaarts A.L., Glajch K.E., Libson E.G., Cahill M.E., Bigio E., Berry R.W. (2007). Phosphorylation and cleavage of tau in non-AD tauopathies. Acta Neuropathol..

[84] Weingarten M.D., Lockwood A.H., Hwo S.Y., Kirschner M.W. (1975). A protein factor essential for microtubule assembly. Proc. Natl. Acad. Sci. U. S. A..

[85] Haj-Yahya M., Gopinath P., Rajasekhar K., Mirbaha H., Diamond M.I., Lashuel H.A. (2020). Site-specific hyperphosphorylation inhibits, rather than promotes, tau fibrillization, seeding capacity, and its microtubule binding. Angew. Chem. Int. Ed. Engl..

[86] Cohen T.J., Guo J.L., Hurtado D.E., Kwong L.K., Mills I.P., Trojanowski J.Q. (2011). The acetylation of tau inhibits its function and promotes pathological tau aggregation. Nat. Commun..

[87] Cleveland D.W., Hwo S.Y., Kirschner M.W. (1977). Purification of tau, a microtubule-associated protein that induces assembly of microtubules from purified tubulin. J. Mol. Biol..

[88] Breuzard G., Hubert P., Nouar R., De Bessa T., Devred F., Barbier P. (2013). Molecular mechanisms of Tau binding to microtubules and its role in microtubule dynamics in live cells. J. Cell. Sci..

[89] Black M.M., Slaughter T., Moshiach S., Obrocka M., Fischer I. (1996). Tau is enriched on dynamic microtubules in the distal region of growing axons. J. Neurosci..

[90] Amniai L., Barbier P., Sillen A., Wieruszeski J.M., Peyrot V., Lippens G. (2009). Alzheimer disease specific phosphoepitopes of Tau interfere with assembly of tubulin but not binding to microtubules. FASEB J..

[91] Alonso A.C., Zaidi T., Grundke-Iqbal I., Iqbal K. (1994). Role of abnormally phosphorylated tau in the breakdown of microtubules in Alzheimer disease. Proc. Natl. Acad. Sci. U. S. A..

[92] Preuss U., Biernat J., Mandelkow E.M., Mandelkow E. (1997). The ‘jaws' model of tau-microtubule interaction examined in CHO cells. J. Cell. Sci..

[93] Mukrasch M.D., von Bergen M., Biernat J., Fischer D., Griesinger C., Mandelkow E. (2007). The "jaws" of the tau-microtubule interaction. J. Biol. Chem..

[94] Gustke N., Trinczek B., Biernat J., Mandelkow E.M., Mandelkow E. (1994). Domains of tau protein and interactions with microtubules. Biochemistry.

[95] Tiernan C.T., Combs B., Cox K., Morfini G., Brady S.T., Counts S.E. (2016). Pseudophosphorylation of tau at S422 enhances SDS-stable dimer formation and impairs both anterograde and retrograde fast axonal transport. Brain. Res. Bull..

[96] Maeda S., Sahara N., Saito Y., Murayama S., Ikai A., Takashima A. (2006). Increased levels of granular tau oligomers: an early sign of brain aging and Alzheimer's disease. Neurosci. Res..

[97] Swanson E., Breckenridge L., McMahon L., Som S., McConnell I., Bloom G.S. (2017). Extracellular tau oligomers induce invasion of endogenous tau into the somatodendritic compartment and axonal transport dysfunction. J. Alzheimers. Dis..

[98] Lasagna-Reeves C.A., Castillo-Carranza D.L., Sengupta U., Clos A.L., Jackson G.R., Kayed R. (2011). Tau oligomers impair memory and induce synaptic and mitochondrial dysfunction in wild-type mice. Mol. Neurodegener.

[99] Jiang L., Lin W., Zhang C., Ash P.E.A., Verma M., Kwan J. (2021). Interaction of tau with HNRNPA2B1 and N(6)-methyladenosine RNA mediates the progression of tauopathy. Mol. Cell..

[100] Hill E., Karikari T.K., Moffat K.G., Richardson M.J.E., Wall M.J. (2019). Introduction of tau oligomers into cortical neurons alters action potential dynamics and disrupts synaptic transmission and plasticity. eNeuro.

[101] Fa M., Puzzo D., Piacentini R., Staniszewski A., Zhang H., Baltrons M.A. (2016). Extracellular tau oligomers produce an immediate impairment of LTP and memory. Sci. Rep..

[102] Wolozin B.L., Pruchnicki A., Dickson D.W., Davies P. (1986). A neuronal antigen in the brains of Alzheimer patients. Science.

[103] Liu K., Liu Y., Li L., Qin P., Iqbal J., Deng Y. (2016). Glycation alter the process of Tau phosphorylation to change Tau isoforms aggregation property. Biochim. Biophys. Acta..

[104] Combs B., Voss K., Gamblin T.C. (2011). Pseudohyperphosphorylation has differential effects on polymerization and function of tau isoforms. Biochemistry.

[105] Chakraborty P., Riviere G., Hebestreit A., de Opakua A.I., Vorberg I.M., Andreas L.B. (2023). Acetylation discriminates disease-specific tau deposition. Nat. Commun..

[106] Voss K., Gamblin T.C. (2009). GSK-3beta phosphorylation of functionally distinct tau isoforms has differential, but mild effects. Mol. Neurodegener.

[107] Permanne B., Sand A., Ousson S., Neny M., Hantson J., Schubert R. (2022). O-GlcNAcase inhibitor ASN90 is a multimodal drug candidate for tau and alpha-synuclein proteinopathies. ACS Chem. Neurosci..

[108] Lane-Donovan C., Boxer A.L. (2024). Disentangling tau: one protein, many therapeutic approaches. Neurotherapeutics.

[109] Congdon E.E., Ji C., Tetlow A.M., Jiang Y., Sigurdsson E.M. (2023). Tau-targeting therapies for Alzheimer disease: current status and future directions. Nat. Rev. Neurol..

[110] Bartolome-Nebreda J.M., Trabanco A.A., Velter A.I., Buijnsters P. (2021). O-GlcNAcase inhibitors as potential therapeutics for the treatment of Alzheimer's disease and related tauopathies: analysis of the patent literature. Expert Opin. Ther. Pat..

[111] Rockenstein E., Overk C.R., Ubhi K., Mante M., Patrick C., Adame A. (2015). A novel triple repeat mutant tau transgenic model that mimics aspects of pick's disease and fronto-temporal tauopathies. PLoS One.

[112] Ryan J.M., Quattropani A., Abd-Elaziz K., den Daas I., Schneider M., Ousson S. (2018). Phase 1 study in healthy volunteers of the O-GlcNAcase inhibitorASN120290 as a novel therapy for progressive supranuclear palsy and related tauopathies [abstract O1-12-05]. Alzheimers Dement.

[113] Kielbasa W., Phipps K.M., Tseng J., Natanegara F., Cheng E., Monk S.A. (2020). A single ascending dose study in healthy volunteers to assess the safety and PK of LY3372689, an inhibitor of O-GlcNAcase (OGA) enzyme. Human/human trials: anti-tau. Alzheimers Dement.

[114] Shcherbinin S., Kielbasa W., Dubois S., Lowe S.L., Phipps K.M., Tseng J. (2020). Brain target occupancy of LY3372689, an inhibitor of the O-GlcNAcase (OGA) enzyme: translation from rat to human: neuroimaging/evaluating treatments. Alzheimers Dement.

[115] Kielbasa W., Shcherbinin S., Goldsmith P., Phipps K.M., Biglan K., Mancini M. (2021). Brain target occupancy of LY3372689, an inhibitor of the O-GlcNAcase (OGA) enzyme, following administration of single and multiple doses to healthy volunteers. Alzheimers Dement.

[116] Lowe S.L., Goldsmith P., Phipps K.M., Kevin D.B., Biglan K., Mancini M. (2021). Single and multiple ascending dose studies in healthy volunteers to assess the safety and PK of LY3372689, an inhibitor of the O-GlcNAcase (OGA) enzyme. Alzheimers Dement.

[117] Kielbasa W., Goldsmith P., Donnelly K.B., Nuthall H.N., Shcherbinin S., Fleisher A.S. (2024). Discovery and clinical translation of ceperognastat, an O-GlcNAcase (OGA) inhibitor, for the treatment of Alzheimer's disease. Alzheimers Dement (N Y).

[118] Yu Y., Zhang L., Li X., Run X., Liang Z., Li Y. (2012). Differential effects of an O-GlcNAcase inhibitor on tau phosphorylation. PLoS One.

[119] Yan X., Zheng J., Ren W., Li S., Yang S., Zhi K. (2024). O-GlcNAcylation: roles and potential therapeutic target for bone pathophysiology. Cell Commun. Signal.

[120] Alonso J., Schimpl M., van Aalten D.M. (2014). O-GlcNAcase: promiscuous hexosaminidase or key regulator of O-GlcNAc signaling?. J. Biol. Chem..

[121] Okamura N., Harada R., Ishiki A., Kikuchi A., Nakamura T., Kudo Y. (2018). The development and validation of tau PET tracers: current status and future directions. Clin. Transl. Imaging.

[122] Combs B., Tiernan C.T., Hamel C., Kanaan N.M. (2017). Production of recombinant tau oligomers in vitro. Methods. Cell. Biol..

[123] Porzig R., Singer D., Hoffmann R. (2007). Epitope mapping of mAbs AT8 and Tau5 directed against hyperphosphorylated regions of the human tau protein. Biochem. Biophys. Res. Commun..

[124] LoPresti P., Szuchet S., Papasozomenos S.C., Zinkowski R.P., Binder L.I. (1995). Functional implications for the microtubule-associated protein tau: localization in oligodendrocytes. Proc. Natl. Acad. Sci. U. S. A..

[125] Yang S., Wang Y., Mann M., Wang Q., Tian E., Zhang L. (2021). Improved online LC-MS/MS identification of O-glycosites by EThcD fragmentation, chemoenzymatic reaction, and SPE enrichment. Glycoconj. J..

[126] Miller R.M., Millikin R.J., Rolfs Z., Shortreed M.R., Smith L.M. (2023). Enhanced proteomic data analysis with MetaMorpheus. Methods. Mol. Biol..

[127] Li Q., Shortreed M.R., Wenger C.D., Frey B.L., Schaffer L.V., Scalf M. (2017). Global post-translational modification discovery. J. Proteome Res..

[128] Millikin R.J., Solntsev S.K., Shortreed M.R., Smith L.M. (2018). Ultrafast peptide label-free quantification with FlashLFQ. J. Proteome Res..

[129] Wang Y., Loomis P.A., Zinkowski R.P., Binder L.I. (1993). A novel tau transcript in cultured human neuroblastoma cells expressing nuclear tau. J. Cell. Biol..

[130] Gamblin T.C., King M.E., Kuret J., Berry R.W., Binder L.I. (2000). Oxidative regulation of fatty acid-induced tau polymerization. Biochemistry.

[131] Gamblin T.C., King M.E., Dawson H., Vitek M.P., Kuret J., Berry R.W. (2000). In vitro polymerization of tau protein monitored by laser light scattering: method and application to the study of FTDP-17 mutants. Biochemistry.

[132] Morris A.M., Watzky M.A., Agar J.N., Finke R.G. (2008). Fitting neurological protein aggregation kinetic data via a 2-step, minimal/"Ockham's razor" model: the Finke-Watzky mechanism of nucleation followed by autocatalytic surface growth. Biochemistry.

[133] Berry R.W., Sweet A.P., Clark F.A., Lagalwar S., Lapin B.R., Wang T. (2004). Tau epitope display in progressive supranuclear palsy and corticobasal degeneration. J Neurocytol.

