# Supplementary material for: O-GlcNAc modification differentially regulates microtubule binding and pathological conformations of tau isoforms in vitro
Source: J Biol Chem. 2025 Feb 3;301(3):108263. doi: 10.1016/j.jbc.2025.108263 (PMC11927755; doi:10.1016/j.jbc.2025.108263)
Supplement: Supporting information [file mmc4.docx]

**Supporting Information: O-GlcNAc modification differentially regulates microtubule binding and pathological conformations of tau isoforms in vitro**

**Alhadidy M.M., Stemmer P.M., Kanaan N.M.**

**Supporting Figure 1.**


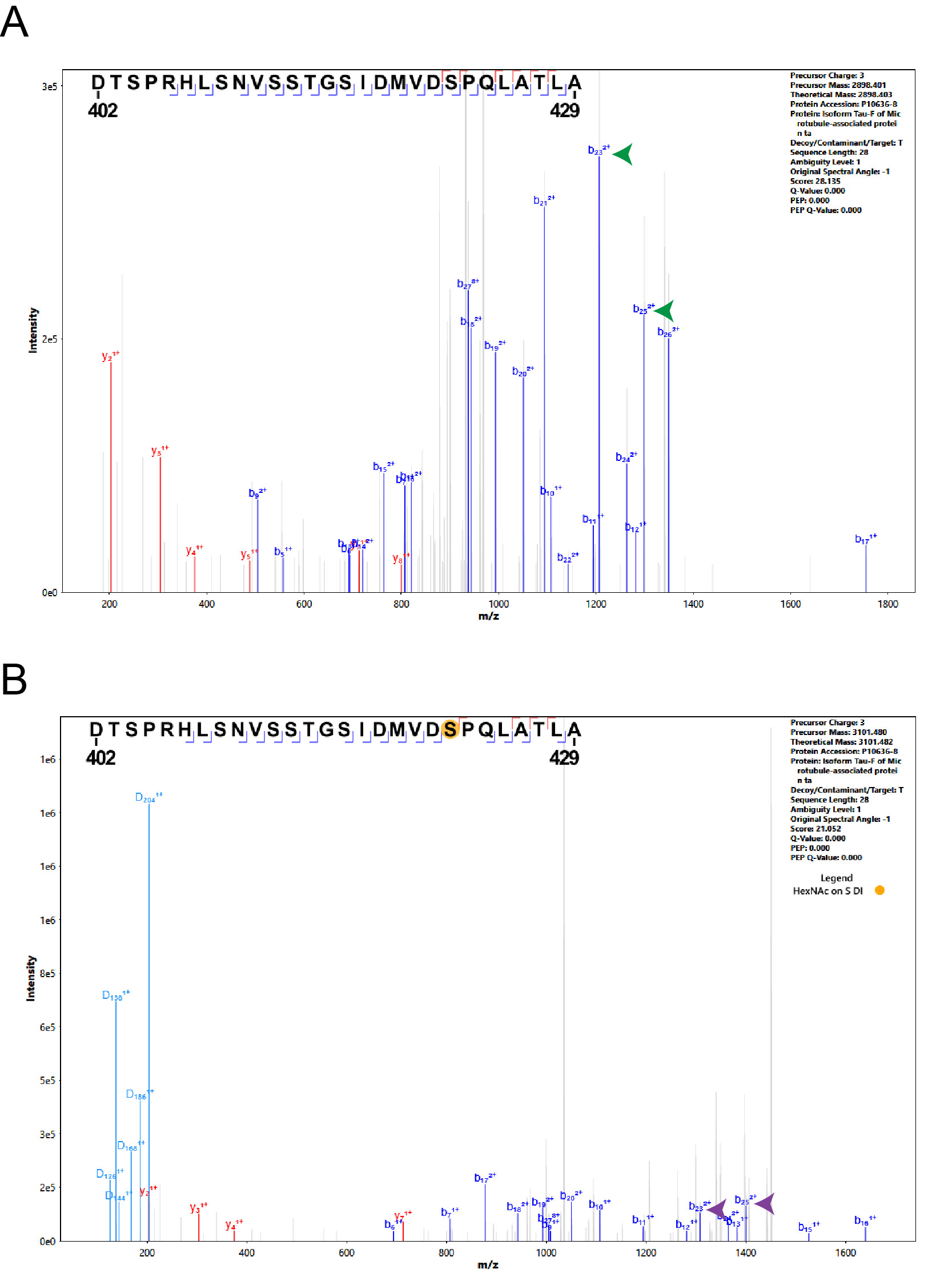


**Figure S1. Sample mass spectra of tau peptides showing O-GlcNAc modification at S422 of hT40.**

A, mass spectrum of peptide spanning amino acids 402-429 in unmodified hT40. Fragmented y-ions showed no change in mass corresponding to O-GlcNAcylation (e.g. b_23_^2+^ m/z = 1206.56 and b_25_^2+^ m/z = 1298.62; green arrowheads). B, mass spectrum of peptide spanning amino acids 402-429 in Glc hT40. Fragmented b-ions show a mass shift corresponding to modification with O-GlcNAc (e.g. b_23_^2+^ m/z = 1308.10 and b_25_^2+^ m/z = 1400.16; purple arrowheads). In addition, all six diagnostic ions produced by O-GlcNAc fragmentation were observed with Glc hT40, but not unmodified hT40. Abbreviations: O-GlcNAc, O-linked-N-acetyl β-d-N-glucosamine; hT40, 2N4R tau isoform; m/z, mass-to-charge ratio; DI, diagnostic ion; D126+, diagnostic ion m/z 126Da; D138+, +, diagnostic ion m/z 138Da; D144+, diagnostic ion m/z 144Da; D168+, diagnostic ion m/z 168Da; D186+, diagnostic ion m/z 186Da D204+, diagnostic ion m/z 204Da.

**Supporting Figure 2.**


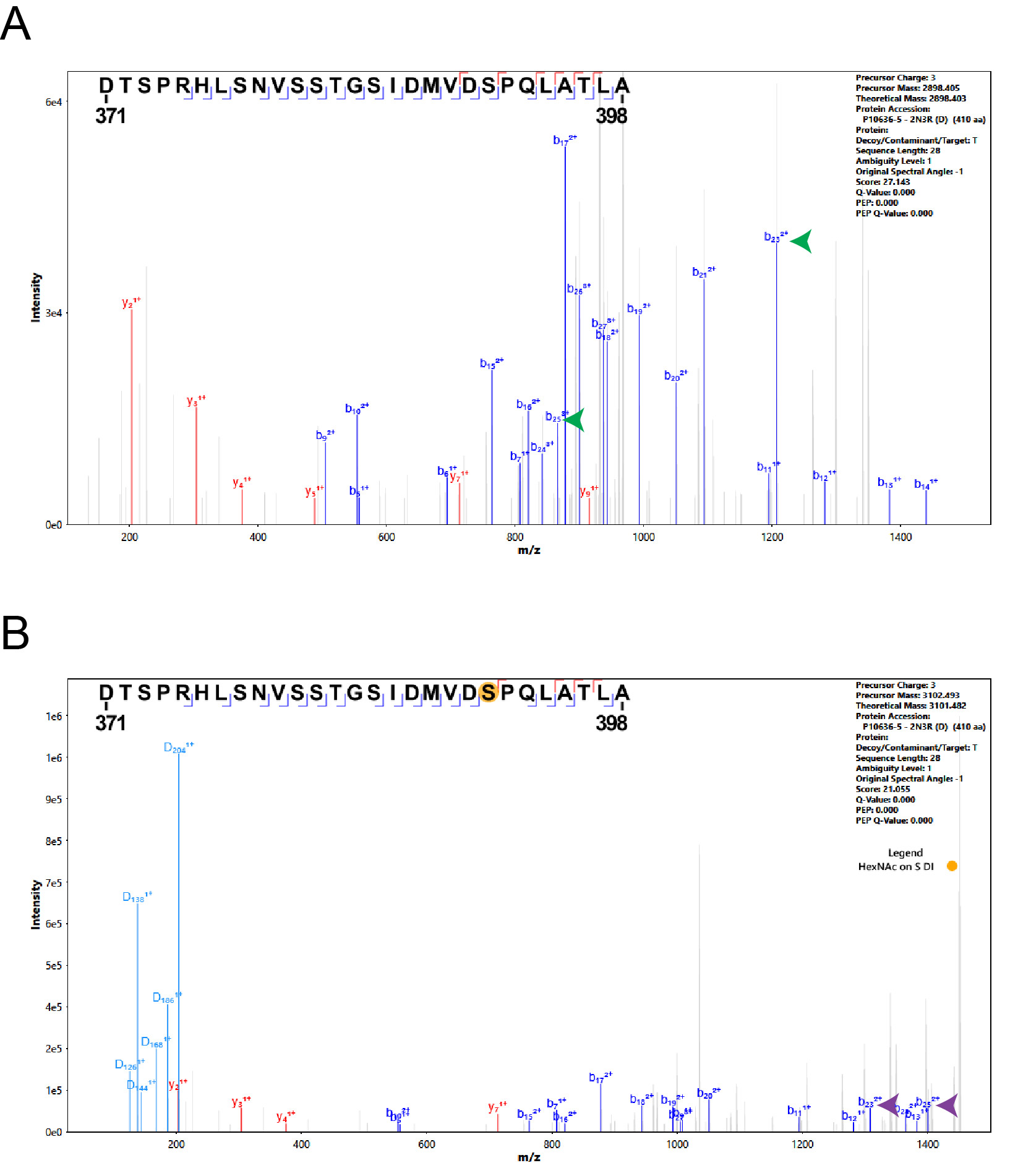


**Figure S2. Sample mass spectra of tau peptides showing O-GlcNAc modification at S422 of hT39.**

A, mass spectrum of peptide spanning amino acids 371-398 in unmodified hT39 (corresponding to 402-429 in hT40). Fragmented y-ions showed no change in mass corresponding to O-GlcNAcylation (e.g. b_23_^2+^ m/z = 1206.56 and b_25_^3+^ m/z = 866.08; green arrowheads). B, mass spectrum of peptide spanning amino acids 371-398 in Glc hT39. Fragmented b-ions show a mass shift corresponding to modification with O-GlcNAc (e.g. b_23_^2+^ m/z = 1308.10 and b_25_^2+^ m/z = 1400.16; purple arrowheads). In addition, all six diagnostic ions produced by O-GlcNAc fragmentation were observed with Glc hT39, but not unmodified hT39. Abbreviations: O-GlcNAc, O-linked-N-acetyl β-d-N-glucosamine; hT40, 2N4R tau isoform; hT39, 2N3R tau isoform; m/z, mass-to-charge ratio; DI, diagnostic ion; D126+, diagnostic ion m/z 126Da; D138+, +, diagnostic ion m/z 138Da; D144+, diagnostic ion m/z 144Da; D168+, diagnostic ion m/z 168Da; D186+, diagnostic ion m/z 186Da D204+, diagnostic ion m/z 204Da.

**Supporting Figure 3.**


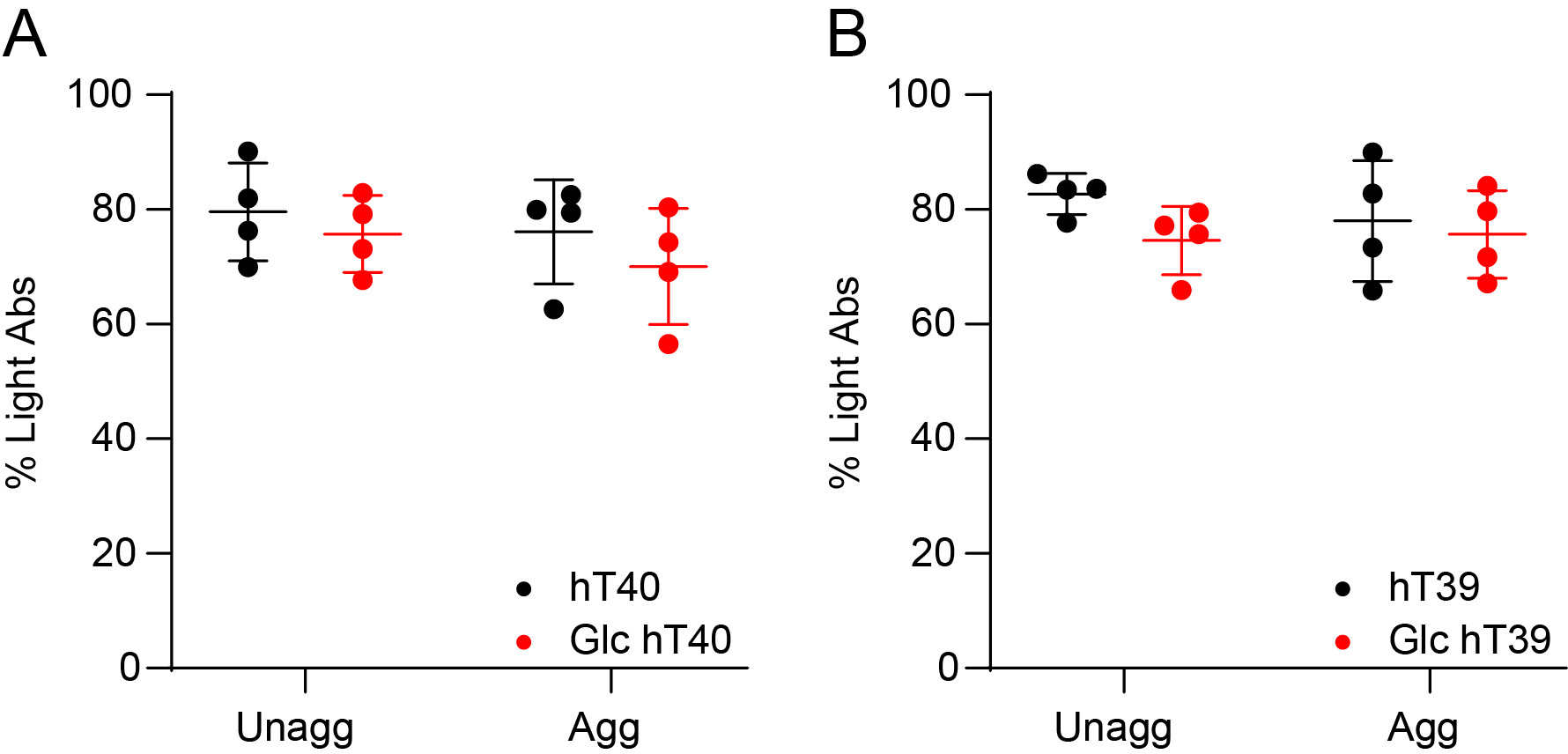


**Figure S3. Sandwich ELISA assay to quantify total tau levels using the Tau13 antibody.**

A, sandwich ELISA assay measuring total tau in unaggregated and aggregated hT40 proteins using Tau13 antibody for capture and R1 antibody for detection. B, sandwich ELISA assay measuring total tau in unaggregated and aggregated hT39 proteins using Tau13 antibody for capture and R1 antibody for detection. Abbreviations: O-GlcNAc, O-linked-N-acetyl β-d-N-glucosamine; hT40, 2N4R tau isoform; hT39, 2N3R tau isoform; R1, tau rabbit polyclonal antibody. Data represented as mean ± SD.
